# TGFβ Drives Metabolic Perturbations during Epithelial Mesenchymal Transition in Pancreatic Cancer: TGFβ Induced EMT in PDAC

**DOI:** 10.3390/cancers13246204

**Published:** 2021-12-09

**Authors:** Meena U. Rajagopal, Shivani Bansal, Prabhjit Kaur, Shreyans K. Jain, Tatiana Altadil, Charles P. Hinzman, Yaoxiang Li, Joanna Moulton, Baldev Singh, Sunil Bansal, Siddheshwar Kisan Chauthe, Rajbir Singh, Partha P. Banerjee, Mark Mapstone, Massimo S. Fiandaca, Howard J. Federoff, Keith Unger, Jill P. Smith, Amrita K. Cheema

**Affiliations:** 1 Department of Oncology, Georgetown University Medical Center, Washington, DC 20057, USA; meena.rajagopal@nih.gov (M.U.R.); sm3451@georgetown.edu (S.B.); yl814@georgetown.edu (Y.L.); jm3330@georgetown.edu (J.M.); bs1126@georgetown.edu (B.S.); sb1886@georgetown.edu (S.B.); 2 Department of Botany, Khalsa College, Amritsar 143002, India; prabhjitkaur@khalsacollege.edu.in (P.K.); sohirajbir@gmail.com (R.S.); 3 Department of Pharmaceutical Engineering & Technology, Indian Institute of Technology, Banaras Hindu University, Varanasi 221005, India; sjain.phe@iitbhu.ac.in; 4 Biomedical Research Group in Gynaecology, Vall Hebron Research Institute (VHIR), Universitat Autònoma de Barcelona, 08035 Barcelona, Spain; altadill@ab-biotics.com; 5 Department of Biochemistry and Molecular and Cellular Biology, Georgetown University Medical Center, Washington, DC 20057, USA; cph51@georgetown.edu (C.P.H.); ppb@georgetown.edu (P.P.B.); 6 Department of Natural Products, National Institute of Pharmaceutical Education and Research (NIPER), Ahmedabad 380054, India; siddheshwarchauthe@gmail.com; 7 Department of Neurology, University of California, Irvine, CA 92697, USA; mark.mapstone@uci.edu (M.M.); mfiandac@uci.edu (M.S.F.); federoff@uci.edu (H.J.F.); 8 Department of Neurological Surgery, University of California, Irvine, CA 92697, USA; 9 Radiation Medicine, Med-Star Georgetown University Hospital, Washington, DC 20057, USA; kru2@georgetown.edu; 10 Department of Medicine, Georgetown University Medical Center, Washington, DC 20057, USA; jps261@georgetown.edu

**Keywords:** tumor microenvironment, pancreatic cancer, TGF beta, 9-cis retinoic acidPANC-1 cells, epithelial mesenchymal transition

## Abstract

**Simple Summary:**

Pancreatic cancer is an aggressive disease with most patients diagnosed at late stages resulting in poor outcomes. While it is known that pancreatic tumor cells undergo epithelial to mesenchymal transition, the metabolic alterations accompanying that transition are not characterized. This study leveraged a metabolomics approach to understand the small molecule and biochemical perturbations that can be targeted for designing strategies for improving outcomes in pancreatic cancer.

**Abstract:**

Pancreatic ductal adenocarcinoma (PDAC) is a highly lethal malignancy wherein a majority of patients present metastatic disease at diagnosis. Although the role of epithelial to mesenchymal transition (EMT), mediated by transforming growth factor beta (TGFβ), in imparting an aggressive phenotype to PDAC is well documented, the underlying biochemical pathway perturbations driving this behaviour have not been elucidated. We used high-resolution mass spectrometry (HRMS) based molecular phenotyping approach in order to delineate metabolic changes concomitant to TGFβ-induced EMT in pancreatic cancer cells. Strikingly, we observed robust changes in amino acid and energy metabolism that may contribute to tumor invasion and metastasis. Somewhat unexpectedly, TGFβ treatment resulted in an increase in intracellular levels of retinoic acid (RA) that in turn resulted in increased levels of extracellular matrix (ECM) proteins including fibronectin (FN) and collagen (COL1). These findings were further validated in plasma samples obtained from patients with resectable pancreatic cancer. Taken together, these observations provide novel insights into small molecule dysregulation that triggers a molecular cascade resulting in increased EMT-like changes in pancreatic cancer cells, a paradigm that can be potentially targeted for better clinical outcomes.

## 1. Introduction

Pancreatic ductal adenocarcinoma (PDAC) has been projected to become the second most common lethal cancer by 2030 [1]. The poor prognosis and less than 10% survival rate of PDAC is mainly attributed to late diagnosis due to the lack of sensitive and specific biomarkers that can be leveraged for early detection [2,3]. Despite the high prevalence of KRAS mutations in pancreatic cancer, therapeutic strategies targeting KRAS have thus far been ineffective. Additionally, treatments with anti-proliferative drugs such as gemcitabine in PDAC have limited effectiveness in part due to the dense fibrosis of the pancreatic tumor microenvironment (TME) and because of the ability of the tumor cells to undergo early epithelial mesenchymal transition (EMT) [4]. Multiple studies suggest that targeting the transforming growth factor-β (TGFβ) pathway may be a viable therapeutic approach [5]. TGFβ is a well-known inducer of EMT that is hyperactive in PDAC. EMT is reported to be essential for the dissemination of cancer cells resulting in metastasis in PDAC [6,7]. The role of TGFβ in late stage PDAC as a tumor promoter is well documented; hence, blocking this signaling pathway is an attractive target for developing more effective therapeutics [8]. However, the role of TGFβ in early PDAC remains ambiguous since there are conflicting reports that suggests its dual nature as both a tumor promoter and suppressor [9,10]. In addition, TGFβ-mediated signaling is known to induce alterations in the tumor microenvironment resulting in desmoplasia [11]. Desmoplasia results from increased proliferation of pancreatic stellate cells (PSC) in the tumor microenvironment along with concomitant changes in other extracellular matrix (ECM) components [12,13]. The subsequent increase in tumor interstitial fluid pressure along with narrowing of the blood vessels makes the delivery of chemotherapeutic agents at the tumor site a daunting task and contributes to overall chemoresistance [14]. Taken together, EMT induced aggressiveness and desmoplasia hinder effective treatment of PDAC patients resulting in chemoresistance and disease relapse [15].

Given the pivotal role played by EMT in PDAC, our goal was to identify metabolic changes associated with EMT in pancreatic cancer that would correlate with tumor progression and metastasis. Hence, we performed global metabolomic profiling of PANC-1 cells treated with TGFβ using UPLC-ESI-QTOF-MS (Ultra performance Liquid Chromatography–Electrospray Ionization Quadrupole-Time of Flight Mass Spectrometry) to identify robust changes in multiple metabolic pathways. Specifically, TGFβ treatment resulted in an accumulation of oncometabolites including those that have been reported to promote tumor invasion in PANC-1 cells. We also found an increase in intracellular levels of retinoic acid that seemed to cause an increase in the expression levels of major components of ECM proteins such as fibronectin (FN) and collagen (COL) as well as upregulation of enzymes involved in protein glycosylation. Taken together, these results, for the first time, suggest that TGFβ-induced EMT is accompanied by metabolic changes and small molecule effectors that impact the tumor microenvironment in a manner that is conducive to tumor growth and invasiveness in pancreatic cancer.

## 2. Materials and Methods

Reagents and chemicals for mass spectrometry: All LC-MS grade solvents including chloroform, ACN, water and methanol were purchased from Fisher Optima grade, Fisher Scientific (Hampton, NJ, USA). High purity formic acid (99%) was purchased from Thermo-Scientific (Rockford, IL, USA). Ammonium formate, debrisoquine and 4-nitrobenzoic acid (4-NBA) were purchased from Sigma-Aldrich (St. Louis, MO, USA). TGFβ, 9-*cis* retinoic acid and all *trans*-retinoic acid were purchased from R&D systems. Internal standards for all *trans* retinoic acid (D5) and adenosine (D2) were purchased from Cambridge Isotope Laboratories and standards. Acetyl CoA was purchased from Cayman Chemical Company (Ann Arbor, MI, USA).

### 2.1. Cell Lines and Culture Conditions

All pancreatic cancer cell lines used in the study were obtained from the Tissue Culture Shared Resource in Lombardi Comprehensive Cancer Center. PANC-1 cells were cultured in Dulbecco’s Modified Eagle Medium (DMEM) supplemented with 10% FBS, 1% l-glutamine, 1% sodium pyruvate, 1% non-essential amino acids, 0.5% fungizone and 1% penicillin–streptomycin [16]. Panc02.13 cells were cultured in Roswell Park Memorial Institute (RPMI-1640) media containing 10% FBS, 1% l-glutamine, 1% sodium pyruvate, 1% non-essential amino acids, 0.5% fungizone, 1% penicillin–streptomycin, 0.2% insulin and gentamycin. ASPC1 and SW1990 cells were cultured in phenol red-free Improved Minimum Essential Medium (IMEM; Invitrogen) containing 10% fetal bovine serum (Atlanta Biologicals), 2 mM L-glutamine, 100 U/mL penicillin G sodium and 100 μg/mL streptomycin sulfate (Sigma). All cell lines used were tested and authenticated at the Tissue Culture Shared Resource (TCSR) in Lombardi Comprehensive Cancer Center by DNA fingerprinting short tandem repeat analysis (STR). All cells were tested negative for mycoplasma and other contaminants.

Cells were grown in a humidified incubator at 37 °C at 5% CO_2_ until they were about 80% confluent after which the media was removed, and cells were washed with PBS. Fresh media devoid of FBS was added, and the cells were serum starved for 24 h. Subsequently, these were separately treated with TGFβ (10 ng/mL) and 9-*cis* retinoic acid (0.5 µM and 1µM) containing fresh serum-free media. All treatments were carried out for 48 h after which cells were harvested by scraping in PBS solution. The cell suspension was centrifuged at 1500 rpm for 20 min at 4 °C, supernatant was discarded and cell pellet was stored at −80 °C until further analysis.

### 2.2. UPLC-ESI-QTOF MS Analysis

For metabolite extraction, the cell pellets were resuspended in 100 μL of water. The tubes were placed on dry ice for 30 sec followed by 90 sec incubation in a 37 °C water bath and the freeze–thaw cycle was repeated three times. The samples were sonicated for 30 sec. An amount of 600 μL of 50% chilled methanol containing internal standards (10 μL of debrisoquine (1 mg/mL in water) and 50 μL of 4-NBA (1 mg/mL in methanol) per 10 mL) was added to the cell pellets. The supernatant was transferred to a fresh tube, and 350 μL chilled 100% ACN was added. In addition, 2 μL of the supernatant was set aside for protein quantification by using the Bradford method [17]. The tubes containing supernatant and ACN were vortexed, incubated on ice for 15 min and centrifuged at 13,000 rpm at 4 °C for 15 min. The supernatant was transferred to a fresh tube and dried under vacuum. The samples were resuspended in 200 μL of solvent A (98% water and 2% ACN) and transferred to MS vial for UPLC-ESI-Q-TOF-MS analysis. An amount of 20 µL from each sample was mixed to generate a pooled QC sample. The sample queue was randomized to avoid bias. Two microliters of each sample was injected onto a reverse-phase 2.1 × 50 mm Acquity 1.7 µm C18 column (Waters Corporation, Milford, MA, USA) using an Acquity UPLC system with a gradient mobile phase consisting of 2% acetonitrile in water containing 0.1% formic acid (solution A) and 2% water in acetonitrile containing 0.1% formic acid (solution B). Each sample was resolved for 10 min at a flow rate of 0.5 mL/min. The gradient consisted of 100% A for 0.5 min, then a ramp of curve 6 to 60% B from 0.5 min to 4.0 min, then a ramp of curve 6 to 100% B from 4.0–8.0 min, held at 100% B until 9.0 min and then a ramp of curve 6 to 100% A from 9.0 min to 9.2 min, followed by a hold at 100% A until 10 min. The column eluent was introduced directly into the mass spectrometer by electrospray. Mass spectrometry was performed on a Q-TOF instrument (QTOF Premiere, Waters, Columbia, MD, USA) operating in either negative (ESI−) or positive (ESI+) electrospray ionization mode with a capillary voltage of 3200 V and a sampling cone voltage of 20 V in negative mode and 35 V in positive mode. The desolvation gas flow was set to 800 L/h, and the temperature was set to 350 °C. The cone gas flow was 25 L/h, and the source temperature was 120 °C. Data were acquired in centroid mode from 50 to 850 m/z in MS scanning. Accurate mass was maintained by infusion of LockSpray interface with Leucine Enkaphalin (556.2771 [M + H]^+^ and 554.2615 [M − H]^−^). Pooled quality control (QC) samples for each matrix comprised an aliquot of all samples in the study set, thus representing a universal set of metabolites. Initially the column was conditioned using this QC sample, and thereafter it was injected after every ten injections to account for reproducibility of the LC-MS data. In addition, a blank and a MetMix standard were randomly injected at an average interval of 10 samples to monitor mass accuracy, retention time drifts and sample carryover. LC-MS data were pre-processed using the XCMS software [18]. We performed multivariate data analysis using R package [19]. Statistically significant *m*/*z* and FDR adjusted *p*-value ≤ 0.05 were run on the UPLC-QToF instrument in the MS/MS mode. Identities for a subset of metabolites were validated by MS/MS. Human Metabolome Database (www.hmdb.ca (accessed on 18 November 2018)), Madison Metabolomics Consortium Database (mmcd.nmrfam.wisc.edu (accessed on 18 November 2018)), LIPID MAPS (www.lipidmaps.org (accessed on 18 November 2018)), KEGG (www.kegg.jp/kegg (accessed on 18 November 2018)) and Metlin (metlin.scripps.edu (accessed on 18 November 2018)) were used for accurate mass-based putative identification of metabolites. Tandem mass spectrometry was used to putatively identify annotations using fragmentation matching.

### 2.3. Targeted Metabolomics Using 7500 QTRAP

Targeted metabolomics method, developed in-house, was used to quantitate > 500 endogenous metabolites using QTRAP^®^ 7500 LC-MS/MS System (Sciex, MA, USA). For this purpose, 50 μL of PBS was added to the cell pellet, and the sample tube was plunged into dry ice for 30 sec and 37 °C water bath for 90 sec. This cycle was repeated for two more times and then samples were sonicated for 1 min, and 100 μL of chilled isopropanol containing internal standards (debrisoquine and 4-nitrobenzoic acid for positive and negative mode of targeted metabolomics, respectively, and labelled lipid standards for each class for targeted lipidomics) was added. The samples were vortexed for 1 min and kept on ice for 30 min followed by incubation at −20 °C for 2 h for protein precipitation. The samples were centrifuged at 13,000 rpm for 20 min at 4 °C. The supernatant was transferred to MS vial for LC-MS analysis. An amount of 20 μL of each prepared sample was mixed to generate the pooled QC sample. An amount of 5 μL of the prepared sample was injected onto a Kinetex 2.6 μm polar C18 100 Å 100 × 3.0 mm (Phenomenex, CA, USA) using SIL-30 AC auto sampler (Shimazdu, Kytoto, Japan) connected with a high flow LC-30AD solvent delivery unit (Shimazdu, Kytoto, Japan) and CBM-20A communication bus module (Shimazdu, Kytoto, Japan) online with QTRAP 7500 (Sciex, MA, USA) operating in positive and negative ion modes. A binary solvent comprising water with 0.1% formic acid (solvent A) and acetonitrile with 0.1% formic acid (solvent B) was used. The extracted metabolites were resolved at 0.2 mL/min flow rate. The LC gradient conditions were as follows: Initial-100% A, 0% B for 2.1 min; 14 min for 5% A and 95% B until 15 min; and 15.1 min for 100% A and 0% B until 20 min. The auto sampler and oven were kept at 15 °C and 30 °C, respectively. Source and gas settings for the method were as follows: curtain gas = 40; CAD gas = 9; ion spray voltage = 1700 V in positive mode and ion spray voltage = 1600 V in negative mode; temperature = 350 °C; ion source gas 1 = 30; and ion source gas 2 = 50.

### 2.4. Targeted Lipidomics Using 7500 QTRAP

A targeted lipidomics method, developed in-house, was used to quantitate 22 classes of lipid metabolites such as diacylglycerols (DAGs), cholesterol esters (CEs), sphingomyelins (SMs), phosphtatidylchloine (PCs), triacylglycerols (TAGs), free fatty acids (FFAs), ceramides (CERs), dihydroceramides (DCERs), hexosylceramides (HCERs), lactosylceramides (LCERs), phosphatidylethnolamines (PEs), lysophosphtatidylchloines (LPCs), lysophosphatidylethnolamines (LPEs), phosphatidylinositols (PIs), lysophosphatidylinositols (LPIs), phosphatidylserines (PSs), phosphatidic acid (PAs), lysophosphatidic acid (LPAs), monoacyl glycerols (MAGs), acylcarnitines (ACs), phosphatidyl glycerols (PGs) and lysophosphatidyl glycerols (LPGs) using QTRAP^®^ 7500 LC-MS/MS System (Sciex, MA, USA). For this purpose, the cell samples prepared above for the targeted metabolomics were used. A binary solvent comprising acetonitrile/water 95/5 with 10 mM ammonium acetate as solvent A and acetonitrile/water 50/50 with 10 mM ammonium acetate as solvent B was used for the resolution. Lipids were resolved on Xbridge amide 3.5 µm, 4.6 × 100 mm (Waters) at 0.7 mL/min flow rate. The LC gradient conditions were as follows: Initial-100% A at 0.2 mL/min, 0% B; 2.0 min at 90.9% A and 9.1% B at 0.2 mL/min; 11.0 min at 50% A and 50% B at 0.7 mL/min; 11.5 min at 30% A and 70% B at 0.7 mL/min; and 12.50 min at 0% A, 100% B at 0.7 mL/min, 15.00 min at 0% A, 100% B at 0.7 mL/min and 15.10 min at 100% A and 0% B at 0.2 mL/min until 17.00 min. The auto sampler and oven were kept at 15 °C and 35 °C, respectively. Source and gas setting were as follows: curtain gas = 45; CAD gas = 9; ion spray voltage = 5200 V in positive mode and ion spray voltage = 4500 V in negative mode; temperature = 350 °C; ion source gas 1 = 45; and ion source gas 2 = 50.

### 2.5. Quality Control and Measures to Monitor Data Quality

The reproducibility and high quality of the LC-MS data were ensured by using several measures. The column was conditioned using pooled QC samples initially, and pooled QC samples were also injected periodically (after every four sample injections) to monitor shifts in signal intensities and retention time as measures of reproducibility and data quality of the LC-MS data. Pooled QC samples were routinely applied to assess and correct for any analytical variance. We also have blank solvent runs between a set of samples (after every 4 samples on either side pooled QC samples) to monitor sample-to-sample carry-over effects. In order to monitor instrumental variance, we also ran an NIST plasma sample (after every 4 samples) prepared in the same manner as actual samples.

### 2.6. Data Processing and Statistical Analysis for QTRAP 7500 Metabolomics and Lipidomics Data

Abundance measurement for metabolites was expressed as intensity units (area under the curve) that were initially normalized to internal standards and processed using Sciex OS 2.0.1 (Sciex). The data were pre-processed using a signal/noise ratio > 20:1 and retention time (RT) tolerance of 5 s after manually checking of metabolites peak to find the reliable features. We also use 20% of missing values in each feature as filter out criteria. Missing values were imputed by half of the minimum positive value in the original data. Thereafter, we used 20% of the coefficient of variation (CV) as our filter criteria to remove any possible noises before data normalization. Analytical drifts (if any) were corrected by quality control based robust LOESS signal correction (QC-RLSC) [20]. All analyses were performed using R package (v 4.0.3). The normalized LC-MS data were log transformed and Pareto scaled. For the 12 samples in the study set, the level of differential expression for each metabolite was calculated using an unpaired t-test, comparing control vs. TGFβ treated, and constrained by FDR corrected *p*-value < 0.05. Figures were created using R and BioRender (www.BioRender.com) accessed on 21 June 2021.

### 2.7. Reverse Transcription-Polymerized Chain Reaction (RT-PCR)

Reverse-Transcription Reaction was performed following the standard protocol of RT^2^ First Strand Kit (Qiagen) and was analyzed by real time PCR. qRT-PCR on EMT related genes, genes involved in glycosylation and genes involved in glucose transport and metabolism were performed according to manufacturer’s protocol, and they are described in detail in Supplementary Materials. The data were analyzed based on ΔΔCt method as per the instructions of the manufacturer.

### 2.8. Ultra-Performance Liquid Chromatography-Multiple Reaction Monitoring-Mass Spectrometry (UPLC-MRM-MS) Based Targeted Metabolomics

#### 2.8.1. MRM-MS Quantitation of Intra-Cellular Levels of Retinoic Acid and Amino Acids in PANC-1 Cells

PANC-1 cells were grown in DMEM, were treated with 10 ng/mL of TGFβ or DMSO for 48 h and harvested as described earlier. Retinoic acid was extracted using a slightly modified protocol as previously described by Kane et al. [21]. Amino acids were extracted using the commercially available protocol for Absolute *IDQ* p180 kit (*Biocrates* Innsbruck) that involves steps for protein removal, internal standard normalization and derivatization using phenylisothiocyanate. Measurements were made in biological triplicates for each treatment; six calibration standards and three quality control samples were integrated in the analyses. All samples were resolved on a UPLC system online with a triple quadrupole MS (Xevo TQ-S, Waters Corporation) operating in the MRM mode. Quantitation was performed by comparing response values of treated samples to controls (normalized to stably labelled isotope standard for a given metabolite).

#### 2.8.2. MRM-MS Based Quantitation of Metabolites (Acetyl CoA) in Human Plasma Samples

Four groups of samples were used for quantitative analysis of metabolites including 11 normal controls (*n* = 11); stages IA, IB and IIA PDAC (*n* = 12); Intraductal Pancreatic Mucinous Neoplasm (IPMN) (*n* = 10); and pancreatitis (*n* = 11). All plasma samples were prepared using a standard protocol. Detailed protocol of metabolite extraction, LC method employed here is explained in detail in Supplementary Materials, and related figures (Figure S1) are included in Supplementary Data File.

### 2.9. Enzyme Linked Immuno Sorbent Assay (ELISA) of Fibronectin (FN1) and O-Linked N-Acetylglucosamine Transferase (OGT) in Human Plasma Samples

FN1 and OGT levels in plasma were determined using commercially available kits from Affymetrix eBioscience and human OGT ELISA kit from MyBiosource.com according to the manufacturer’s recommendations. For this assay, a total of 39 plasma samples including early stage PDAC (*n* = 11), IPMN (*n* = 9) and acute pancreatitis (*n* = 10) as well as normal controls (*n* = 9) were analyzed. The clinical samples were made available through the Indivumed repository at the MedStar-Georgetown University hospital protocols while the normal controls (*n* = 11) were recruited as a part of the Rochester aging study (RAS) under approved IRB protocols [22]. Clinical and demographic characteristics of the cohort such as age, sex and ethnicity were recorded. All samples were collected under 12 h fasting conditions using stringent procedures for collection and storage, thus minimizing confounding by pre-analytical variables on downstream MS analyses. Other factors and coexisting conditions such as BMI, jaundice, diabetes, smoking and alcohol traits for each subject were also annotated. ELISA results are presented as the mean ± SEM, with the number of experiments indicated in the figure’s legend. Statistical significance was assessed using ANOVA for multiple comparisons with GraphPad Prism (GraphPad Software Inc., San Diego, CA, USA).

## 3. Results

### 3.1. TGFβ Induced EMT in PANC-1 Cells Is Accompanied by Robust Changes in Metabolic Profiles

We confirmed that TFGβ induced an increase in expression of N-cadherin (CDH2) and a decrease in expression levels of E-cadherin (CDH1) at both protein and mRNA levels in accordance with previous reports as hallmarks of EMT induction in PANC-1 cells that carry a mutant KRAS allele [23] (Figure S2A–D). In contrast, there was no significant change in cellular morphology or N-cadherin levels in TGFβ-treated Panc02.13 cells, a KRAS wt pancreatic adenocarcinoma cell line, although a slight decrease in the levels of E-cadherin was observed (Figure S2E–H). Moreover, there was no change in the intracellular levels of retinoic acid upon TGFβ treatment in Panc02.13 cells (Figure S2I).

These results suggest that mutant KRAS may be required for TGFβ mediated induction of EMT in pancreatic cells. Another difference between PANC-1 and Panc02.13 cells is that the former expresses high levels of the cholecystokinin-B receptor (CCK-BR) that is responsible to tumor growth when activated [24] and the Panc02.13 cells lack CCK-BR and only have CCK-AR [25]. We have shown that blockade of the CCK-BR signaling pathway in mice bearing PANC-1 orthotopic tumors with proglumide decreases metastases by inhibiting EMT [26]. The major ligand that activates CCK-BR is gastrin; and TGFB has been shown to activate the gastrin promoter [27].

**Figure 1:**
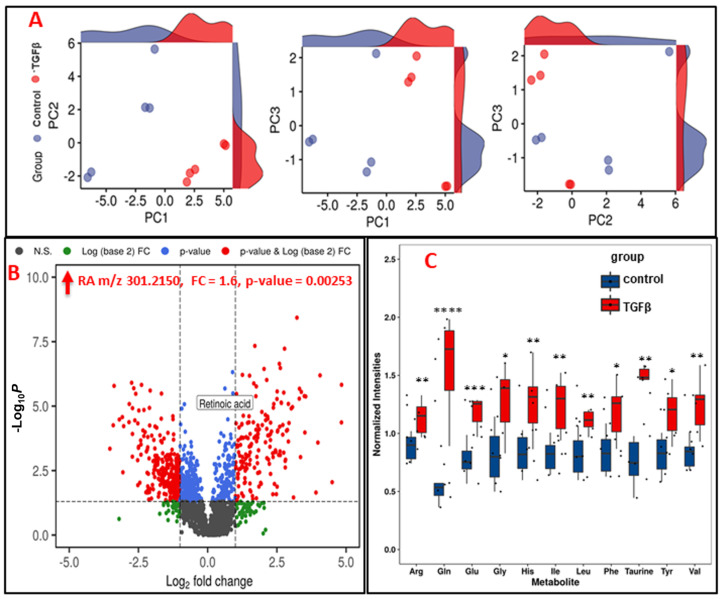
TGFβ treatment of PANC-1 cells induces robust changes in metabolism. (**A**). PCA plot showing the separation between TGFβ and DMSO treated PANC-1 cells based on metabolomics profiles. The *X*-axis shows interclass separation while *Y*-axis illustrates the intra-class variance. (**B**). Volcano plot showing combined visualization of dysregulated metabolites based on fold change on *X*-axis and *p*-value on *Y*-axis. Each dot represents a feature; red colored dots have significant FC (≥0.5 or ≤2.0) and *p*-value (≤0.05). (**C**) Targeted MRM-MS-based quantitative analysis of amino acids that were found to be significantly (FDR ≤ 0.05) dysregulated in PANC-1 cells upon TGFβ treatment. Box and whisker plots representing indicated metabolite levels in PANC-1 cells after TGFβ treatment. * *p*-value < 0.05; ** *p*-value < 0.005; *** *p*-value < 0.0005; **** *p*-value < 0.00005.

In order to delineate metabolic changes concomitant to EMT, we performed untargeted profiling of PANC-1 cells treated with TGFβ. Pre-processing of MS data yielded 2000 and 1500 features in the electrospray (ESI) positive and negative ionization modes, respectively. Inherent differences in metabolic profiles (with and without TGFβ treatment) were visualized using a descriptive principal component analysis (PCA) plot that showed maximal separation between the two comparative groups (Figure 1A). Next, with a fold-change criterion of ≥0.5 or ≤2.0 and *p*-value ≤ 0.05), multiple metabolites were found to be significantly dysregulated upon TGFβ treatment which were visualized as volcano plots (Figure 1B and Table S1). Subsequently, we putatively annotated a subset of metabolites that were significantly dysregulated upon TGFβ treatment using tandem mass spectrometry (MS/MS), which is listed in Table S2. Figure S2J, shows MS/MS validations of a subset of metabolites that were found to be significantly dysregulated in PANC-1 cells upon TGFβ treatment. We found retinoic acid, pseudouridine and prostaglandin E2 to be significantly upregulated in TGFβ-treated PANC-1 cells. Interestingly, reduced glutathione levels were significantly downregulated, suggesting an increase in the oxidative stress status of TGFβ-treated PANC-1 cells, which is a finding that has been reported by other groups [28]. We also observed upregulation of several amino acids in TGFβ treated PANC-1 cells, which was further confirmed via multiple reaction monitoring based mass spectrometry (MRM-MS) (Figure 1C, Table S3).

**Figure 2:**
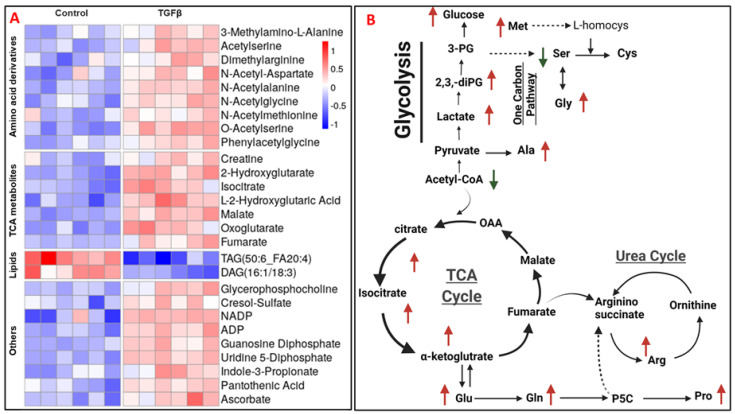
(**A**). Heat map visualization of significantly dysregulated metabolites having FDR ≤ 0.05, corresponding to their relative intensity levels in PANC-1 control and PANC-1 TGFβ treated groups. (**B**). Pathway perturbations in energy and amino acid metabolism in PANC-1 cells following TGFβ treatment.

Next, we performed targeted quantification to confirm other significant metabolic dysregulations in PANC-1 cells upon TGFβ treatment, as observed initially with untargeted profiling. Hence, we quantified a large number of metabolites and lipids in PANC-1 cells treated with TGFβ or DMSO using in-house developed targeted metabolomics (includes >500 small polar molecules) and deep lipidomics (22 classes of lipids) MRM-MS-based panels on a triple quadrupole mass spectrometer (QTRAP^®^ 7500 LC-MS/MS system, Sciex, MA, USA). Data pre-processing was followed by statistical and multivariate analyses that enabled the visualization of distinctive metabolic profiles of PANC-1 treated with TGFβ as compared to control (Table S4). We used hierarchical clustering to examine differential abundance and found robust changes, including significant upregulation of amino acids and their derivatives and TCA metabolites after TGFβ treatment (Figure 2A). Another striking observation was elevation in branched chain amino acids (BCAAs), leucine, isoleucine and valine in accordance with a previously reported study [29].

We also observed significant upregulation of TCA cycle metabolites such as 2-hydroxyglutaric acid, oxoglutarate, isocitrate, malate and fumarate. In addition, the intracellular levels of oxalate, maleic acid, ascorbate and NADP were also found to be upregulated. Most of the metabolic dysregulations observed herein represent interconnected metabolic networks that span central roles in several pathways such as glutaminolysis, the TCA cycle and glycolysis along with amino acid metabolism (Figure 2B).

Dyslipidaemia after TGFβ treatment mainly involved downregulations in the levels of lipid species such as FFAs, LPEs, PSs and TAGs. The downregulated FFAs included myristoleic acid, oleic acid, linoleic acid, dihomo-linoleic acid, arachidonic acid and docosatetraenoic acid (Table S4).

**Figure 3:**
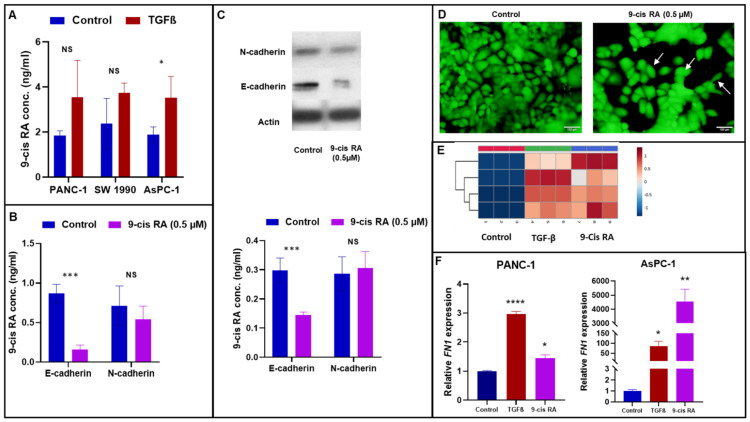
9-cis RA causes a decrease in E-cadherin expression in PANC 1 cells. (**A**). TGFβ treatment in PANC1, SW1990 and ASPC1 cell lines resulted in an increase in intracellular levels of 9-cis Retinoic acid than compared to control treated cells. 9-cis RA was measured using targeted mass spectrometry. (**B**). Gene expression of E-cadherin and N-cadherin in PANC1 cells following 9-cis RA treatment. The bars indicate relative expression of the indicated genes (mean ± SD from three determinations) adjusted with GAPDH. (**C**). Western blot analysis showing relative levels of E-cadherin and N-cadherin after 9-cisRA treatment of PANC1 cells. (**D**). Phase Contrast Microscopy shows altered morphology in 9-cis RA treated PANC1 cells. PANC1 cells were serum starved for 24 h and subsequently treated with 0.5 µM 9-cis RA for 48 h while the controls were treated with DMSO under the same conditions. After 9-cis-RA treatment, PANC1 cells organize to form elongated epithelial structures, similarly to EMT (shown by arrows). (**E**). Heat map of a subset of dysregulated genes in PANC1 analyzed by EMT array in response to treatment with TGFβ and 9-cis RA. Each row on the heat map represents normalized expression for a unique gene. (**F**). Bar graph showing increase in transcript levels of FN1 in PANC-1 and AsPC-1 cells as determined by RTPCR, in response to TGFβ and 9-cis RA treatments, relative to control. FN1 expression within each group was normalized to levels of GAPDH. * *p*-value < 0.05; ** *p*-value < 0.005; *** *p*-value < 0.0005; **** *p*-value < 0.00005.

An interesting and somewhat unexpected observation was a significant increase in the intracellular levels of retinoic acid following TGFβ treatment of PANC-1 cells. We confirmed this finding by using other established pancreatic cancer cell line models by targeted mass spectroscopy (Figure 3A). Given the pivotal role of retinoic acid as a cellular differentiating agent, we sought to understand the role of this small molecule in TGFβ-induced EMT in PANC-1 cells. We used 9-*cis* RA and all *trans*-retinoic acid (atRA) to determine if downstream signaling events, at least in part following TGFβ treatment, were mediated by a particular isomer of RA. Hence, we treated PANC-1 cells with 0.5 µM 9-*cis* RA and 1 µM (at) RA [30]. As shown in Figure 3B,C, treatment of PANC-1 cells with 0.5 µM 9-*cis* RA caused a significant decrease in E-cadherin although there was no increase in N-cadherin. Immunofluorescent microscopy showed significant changes in morphology in 9-*cis* RA treated PANC-1 cells (Figure 3D). Previously, Byers et al. have reported morphological changes in breast cancer cells upon treatment with 9-*cis* RA [31]. On the other hand, (at) RA treated PANC-1 cells showed no significant changes either in cellular morphology or the relative expression of CDH1 and CDH2 (Figure S2K–M), suggesting that 9-*cis* RA was more likely to act as an effector molecule following TGFβ treatment.

Next, we estimated the intracellular levels of 9-*cis* RA in PANC-1 cells upon TGFβ treatment using targeted MRM-MS (MRM transitions detailed in Table S5). The intracellular concentration of retinoic acid after TGFβ treatment was found to be approximately 0.5 µM, which was calculated by extrapolating from a standard calibration curve (Figure S3). All our subsequent treatments were designed to study the role of this small molecule in modulating EMT-mediated metabolic reprogramming; therefore, we used 0.5 µM of 9-*cis* RA for treating PANC-1 cells to mimic intracellular concentrations.

### 3.2. TGFβ and 9-cis RA Treatment Markedly Dysregulates Genes Involved in ECM Remodeling in PANC-1 Cells

One of the intriguing observations in the study thus far was that treatment with 9-*cis* RA caused a partial EMT response (loss of epithelial morphology and downregulation of E-cadherin); however, we did not observe an increase in mesenchymal markers such as N-cadherin or vimentin. We took a multifaceted approach to identify the precise role of 9-*cis* RA in TGFβ-mediated EMT induction. First, we examined the effect of TGFβ and 9-*cis* RA treatments on the expression of a panel of 84 known markers of EMT in PANC-1 cells using a human EMT cDNA array. As expected, treatment with 10 ng/mL of TGFβ elicited a classical and complete EMT response with alterations in the expression levels of genes known to be involved in cell growth and proliferation such as *AKT1* (AKT serine/threonine kinase 1), *BMP7* (bone morphogenetic protein 7), *EGFR* (epidermal growth factor receptor), *ILK* (integrin linked kinase), *MST1R* (macrophage stimulating 1 receptor) and *TGFβ3* (transforming growth factor beta 3), and *SNAI3* (snail family transcriptional repressor 3), *SPARC* (secreted protein acidic and cysteine rich), *KRT14* (keratin 14), *KRT19* (keratin 19) and *TSPAN13* (tetraspanin 13) significantly decreased compared to control. The expression of *PDGFRB* (platelet-derived growth factor receptor beta) that is involved in cell mobility and migration along with *ITGAV* (integrin subunit alpha V) significantly increased after TGFβ treatment. Transcription factors *GSC* (goosecoid homeobox) and *STAT3* (signal transducer and activator of transcription 3) were at least 1.5-fold reduced in TGFβ-treated cells compared to control (Table S6). Moreover, the platelet derived growth factor receptor β (*PDGFRB*) gene that is known to drive EMT mediated metastasis in PDAC [32] was about 8-fold up after treatment with TGFβ. As reported previously, treatment with TGFβ caused upregulation of genes involved in the protein components of the ECM, namely *COL3A* (collagen type 3A, Figure S4) and *FN1* (fibronectin, Figure 3E,F). The observed relative expression of the aforementioned genes after TGFβ treatments compared to control is indicative of the complex mechanisms by which TGFβ induces EMT.

On the other hand, while treatment with 9-*cis* RA significantly increased the expression of ECM component markers such as fibronectin, collagen family and PDGFRB compared to controls (no treatment), it did not trigger a complete EMT induction response. Metalloproteinases (*MMP2* and *MMP9*), known to be involved in the degradation of ECM proteins [33], were significantly downregulated upon 9-*cis* RA treatment compared to TGFβ treatment but were not significantly different from that of controls. We also observed a marked downregulation in the expression of *BMP7*, a known negative regulator of TGFβ-induced EMT [34]. We performed a wound healing assay and measured cell migration both 24 and 48 h post treatment with either 9-cis RA or TGFβ. In both PANC-1 and AsPC-1 cells, we found a significant increase in the migratory potential of cells at both time points after treatment with either TGFβ or 9-cis RA (Figure S5). Overall, treatment with 9-cis RA induced significant change in PANC-1 and AsPC-1 cells even without inducing significant changes in all known markers of EMT. These results suggest that 9-cis RA and TGFβ treatments of pancreatic cancer cells have functional consequences. However, the changes induced by 9-cis RA and TGFβ do have functional consequences [35].

### 3.3. TGF β and 9-cis RA Promote EMT Induced O-Glycosylation

**Figure 4:**
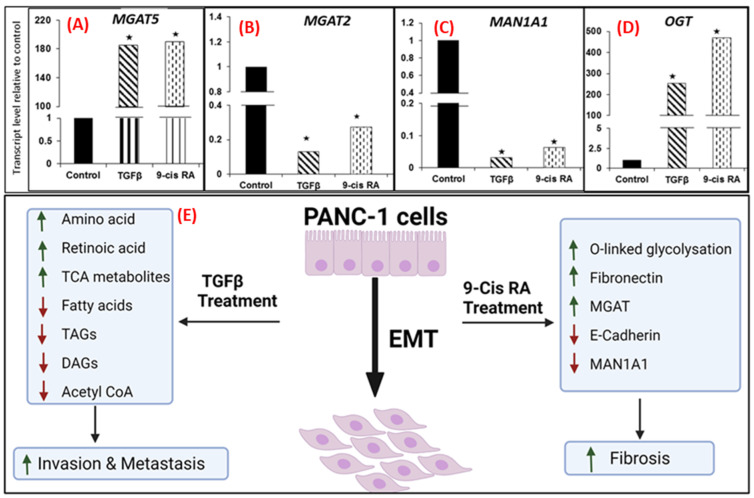
TGFβ and RA trigger metabolic dysreulations characteristic of an invasive metabotype. (**A**–**D**). TGF beta and 9-cis RA induce robust changes in the expression of genes involved in protein glycosylation. Relative expression of *MGAT5*, *MGAT2*, *MAN1A1* and *OGT* in PANC-1 cells was performed using Taqman qRT-PCR method. Each bar shows transcript level of the mentioned genes in PANC-1 cells after treatments (TGFβ and 9-*cis* RA) relative to control after normalized to housekeeping genes. Data represent mean ± SE from duplicate samples (* *p* ≤ 0.05). (**E**). Proposed model illustrating metabolic and gene expression alterations following TGFβ and 9-*cis* RA treatments in PANC-1 cells. TGFβ treatment caused an increase in intracellular retinoic acid levels and changes in amino acid, TCA metabolite and lipid profiles were also observed which may result in increased invasion and metastasis. 9-*cis* RA treatment alone decreased expression of E-cadherin with a concomitant increase in the levels of ECM protein such as FN1 resulting in likely contributions to increased fibrosis.

Our studies thus far demonstrated changes in expression patterns of protein components of ECM predominantly FN1 upon 9-*cis* RA treatment. FN1 is an abundant ECM glycoprotein that orchestrates the assembly of other ECM components including collagen to provide a scaffold for cellular adhesion. Glycosylation of ECM proteins contributes to a dense stroma that is a hallmark of the tumor microenvironment in pancreatic cancer [36]. In order to determine whether enzymes mediating protein glycosylation are modulated by TGFβ and 9-*cis* RA treatment, we, therefore, performed gene expression analysis for an array of glycosylation enzymes. We found dysregulated expression of several genes including monoacylglycerol acyltransferase (*MGAT*) family and mannosyl oligosaccharide glucosidase (*MOGS*) and α-glucosidase I upon TGFβ and 9-*cis* RA treated PANC-1 cells (Figure 4A–C). We also observed increased expression of O-linked N-acetylglucosamine transferase (*OGT*) upon TGFβ and 9-*cis* RA treatment in PANC-1 cells, indicating their putative role in increased glycosylation (Figure 4D). Taken together, these findings suggest a possible role of RA signaling pathway in modulating increased fibrosis in PDAC (Figure 4E); this is a novel finding that warrants further investigations with a goal of developing improved therapeutic strategies that target desmoplasia in PDAC.

### 3.4. Plasma Fibronectin, OGT and Acetyl CoA Levels Are Significantly Increased in Patients Diagnosed with Early Stage Pancreatic Cancer

**Figure 5:**
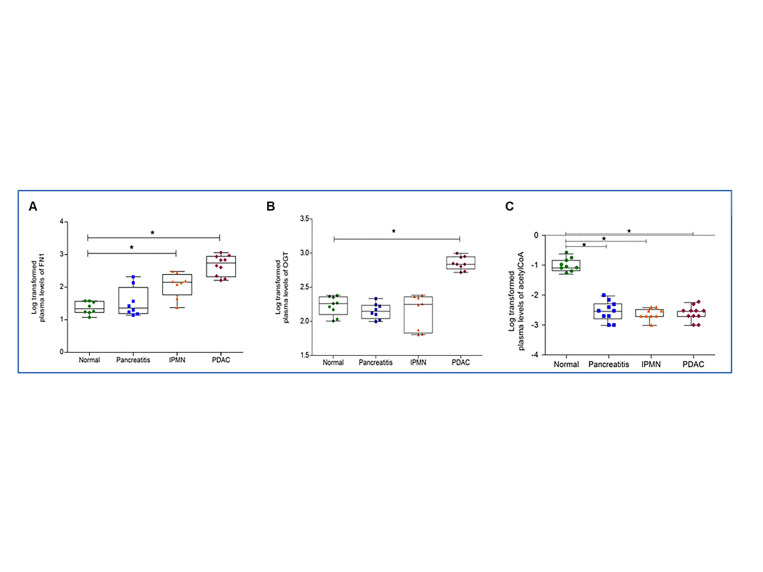
Increased levels of fibronectin, OGT and acetyl CoA in patient plasma samples correlate with pancreatic disease phenotype. FN1 (**A**) and OGT (**B**) levels in human plasma from patients with normal controls (*n* = 8), chronic pancreatitis (*n* = 8), IPMN (*n* = 8) as well as plasma from patients diagnosed with early stage PDAC (*n* = 10) as measured by ELISA. (**C**). Plasma levels of acetyl CoA in 11 PDAC, 9 IPMN, 10 pancreatitis and 9 normal samples as determined by UPLC-MRM MS. Target Lynx was used to obtain the response values for the metabolite in each sample, which was log transformed, and statistical analysis was carried out using GraphPad prism (* *p* ≤ 0.05).

In order to ascertain clinical significance of the aforementioned findings, we used quantitative ELISA to determine the levels of fibronectin and OGT in plasma obtained from a well-defined cohort of early stage PDAC patients. Clinical and demographic characteristics of the cohort as age, sex and ethnicity are detailed in Table S7. Plasma levels of FN1 were significantly higher in PDAC patients (572.85 μg/mL): a 23-fold increase when compared to controls (25.03 μg/mL) (Figure 5A) and about 4-fold to 10-fold higher than compared to pancreatitis patients (59.2 μg/mL) and IPMN patients (135.94 μg/mL), respectively. Interestingly, plasma OGT levels (Figure 5B) were also significantly elevated in PDAC patients (724.4 ng/mL) compared to controls (175.8 ng/mL); however, there was no significant difference in mean values of OGT plasma concentrations between IPMN, pancreatitis and normal groups. We explored the relationship between FN1 and OGT levels and outcomes in the plasma of six PDAC patients in which clinical data were available. There was a modest correlation between FN1 level and time to progression; however, there was no correlation between FN1 and survival or OGT levels and outcome (Figure S6). The plasma upregulation of FN1 and OGT levels in PDAC patients can be potentially used as a prognostic marker of PDAC. Moreover, the plasma levels of acetyl CoA were found to be downregulated in clinical samples (Figure 5C), which may be corelated with our findings of an increase in TCA cycle metabolites and glycolytic flux in PANC-1 cells, towards a hypoxic tumor microenvironment.

## 4. Discussion

Epithelial-to-mesenchymal transition (EMT) has been extensively reported to contribute pathologically to fibrosis and cancer progression and reported as a key priming event for tumor development [37]. Pancreatic cancer is characterized by relatively smaller tumor volumes and a dense stroma that makes drug delivery a significant challenge [38]. While TGFβ-mediated EMT in cancer cells is well documented, concomitant metabolic changes remain to be characterized [39]. Hence, the main focus of the study was to delineate metabolic dysregulations accompanying EMT induced by TGFβ treatment in PANC-1 cells. Here, for the first time, using a combinatorial metabolomics/lipidomics profiling approach, we discovered significant metabolic changes in PANC-1 cells upon TGFβ treatment that were subsequently validated by targeted quantitative mass spectrometry.

TGFβ treatment of PANC 1 cells resulted in significant perturbations in an array of biochemical pathways including energy, amino acid and lipid metabolism. For example, we observed a decrease in intracellular levels of acetyl-CoA and a concomitant upregulation in the levels of 2-hydroxyglutarate, fumarate, malate and isocitrate upon TGFβ treatment. This metabolic shift is likely to favour an increase in glycolytic flux conducive to a hypoxic tumor microenvironment. We also observed upregulation of several amino acids including branched chain amino acids (BCAA) (leucine, isoleucine and valine), glutamine and phenylalanine. Aberrant amino acid metabolism and aerobic glycolysis have been shown to play important roles in pancreatic cancer progression [40,41]. Glutamine is involved in multiple biological processes required for cancer cell growth and proliferation such as biosynthesis of glucosamine, purines, pyrimidines, non-essential amino acids and nicotinamide adenine dinucleotide (NAD). Notably, glutamine is an essential component of redox homeostasis and supports tumor growth of PDAC cells in an oncogenic KRAS-driven manner [42]. BCAAs serve as major nitrogen donors for alanine and glutamine synthesis and have an important role in protein synthesis [43]. Leucine, apart from being an important biological factor, also regulates the mammalian target of rapamycin (mTOR) pathway through nutrient signaling [44,45]. The mTOR pathway is recognized as a critical regulator of cellular function since it has roles in cell growth, proliferation and protein translation [46]. We also observed the upregulations of amino acid arginine and tryptophan that have recently been reported as effective targets of tumor immunotherapy as they have roles in the regulation of T-cell survival, proliferation and activation [47]. Finally, we observed significant dyslipidemia upon TGFβ treatment in PANC-1 cells that included downregulation of lipid species such as free fatty acids (FFAs) including myristoleic acid, oleic acid, linoleic acid, dihomo-linoleic acid, arachidonic acid and docosatetraenoic acid, LPEs, PSs and TAGs. Saturated and monounsaturated fatty acids have been shown to promote progression of pancreatic cancer [48]. In contrast, a metabolomics and transcriptomics study showed a decrease in the levels of lipase and a panel of fatty acids in pancreatic tumors [49]. Given the critical roles of metabolic crosstalk between cancer cells and stromal components, targeting metabolic vulnerabilities of TME may, thus, be considered a potentially effective therapeutic approach for PDAC treatment.

A novel finding of this study was the upregulation of 9-*cis* RA upon TGFβ treatment in pancreatic adenocarcinoma cells that harbor a mutant KRAS gene, while these changes were not apparent in Panc02.13 cells. Upregulation of retinoic acid, following TGFβ treatment, was confirmed in multiple pancreatic cancer cell lines using multiple reaction monitoring mass spectrometry. Although retinoic acid signaling is crucial for the developing pancreas and its interaction with other signaling cascades such as Wnt and TGF is well documented [50], the role of 9-*cis* RA signaling in TGFβ induced EMT is hitherto unreported. We found that treatment of PANC-1 and AsPC-1 cells with 9-*cis* RA induces the expression of major ECM proteins including fibronectin (FN1) and Collagen (COL3A). We further showed that 9-cis RA increased the migratory potential of these cells. It is reasonable to assume that increased FN1 levels could be driving EMT in these cells, contributing to the invasiveness in PDAC. Tumor cells have been reported to upregulate the expression of ECM proteins including FN1 to create a pro-tumor stromal environment [51]; however, further investigations are needed to delineate the precise role of 9-*cis* RA in mediating changes in the TME. Interestingly, the observed upregulation of amino acids and energy metabolism may contribute to a shift in to a more proliferative and invasive phenotype [52,53]. Future studies are needed to examine if upregulation of 9-*cis* RA is a generic phenomenon occurring in different cancer cell types undergoing TGFβ-induced EMT. The upregulation of FN1 and COL3A was also confirmed in plasma samples obtained from patients with resectable PDAC. Interestingly, the plasma levels of these ECM proteins were found to increase progressively in patients carrying precursor lesions of pancreas (IPMN) and PDAC as compared to pancreatitis, thus emphasizing the potential utility of these proteins as prognostic markers for pancreatic carcinogenesis [33].

We also observed an upregulation of the enzyme O-GlcNAc transferase (OGT), which is in accordance with literature reports [54]. In order to further investigate glycosylation changes during EMT-related pancreatic cancer aggressiveness, we determined the gene expression of glycosylation enzymes after treatment with TGFβ and 9-*cis* RA. Using quantitative RT-PCR, we identified significant changes in the expression of *MGAT5*, *MGAT2*, *MAN1A2*, *MOGS* and *OGT*. *MGAT* gene codes for N-acetyl glucosaminyl transferases that participates in N-glycosylation within the Golgi [55]. *MGAT5* was significantly upregulated in both TGFβ and 9-*cis* RA treatments of PANC-1 cells. Interestingly, *MGAT5* gene product GNTV is known to play a role in mediating aberrant elongation of branched N-glycans, resulting in the formation of complex tri-antennary and tetra-antennary structures associated with metastatic phenotype [56]. On the contrary, *MGAT2*, an enzyme involved in early branching, is significantly downregulated, and this agrees with reports that *MGAT5* can transfer GlcNAc residues only in the absence of *MGAT2*. Taken together, we observed an alteration in the levels of *MGAT* that is consistent with increased branching of N-glycans. We also observed a decrease in *MAN1A1* levels after both TGFβ and 9-*cis* RA treatments. MAN1A1 is a mannosidase enzyme that is responsible for the cleavage of mannose residues during biosynthesis of N-glycosylation of proteins making way for the addition of other sugar moieties, resulting in the formation of highly variable complex N-glycans. Our results concur with Legler et al., who have reported decreased *MAN1A1* activity along with overexpression of *MGAT5* in breast cancer cells that resulting in high cellular motility and invasiveness of these tumor cells [57]. Several studies have also explored the modulation of N-glycans in leucocyte trafficking and how endothelial cells expressing N-glycans adhere to leucocytes [58,59]. It is possible that tumor cells could use similar mechanisms and adhere to vascular walls, thereby promoting extra vascularization.

In conclusion, these novel findings underscore the role of molecular phenotyping technologies for discovering previously unanticipated pathway perturbations with significant roles in promoting cancer metastasis and fibrosis. Our approach underscores the power of molecular phenotyping approaches that augment the identification of metabolic vulnerabilities of aggressive tumors that can potentially help in the development of novel therapeutics. Identification of molecular targets that can potentially alleviate stromal fibrosis is likely to improve local drug delivery relative to the proliferating tumor, thus impacting the success of therapeutic regimens and clinical outcomes in pancreatic cancer.

## Data Availability

Not applicable.

## Supplementary Materials

The following are available online at https://www.mdpi.com/article/10.3390/cancers13246204/s1, Table S1. Statistical analysis of untargeted profiling data comparing PANC-1 cells treated with either TGFβ or DMSO; Table S2. Full list of tandem MS validated metabolites with details; Table S3. Statistical analysis results for the amino acid quantitation by targeted MRM-MS using UPLC-TQS comparing PANC-1 cells treated either with TGFβ or DMSO; Table S4. List of significantly dysregulated targeted MRM-MS data using QTRAP 7500 comparing PANC-1 cells treated either with TGFβ or DMSO; Table S5. Q1/Q3 transitions for metabolites quantified using multiple reaction monitoring mass spectrometry (MRM-MS); Table S6. The CT values for EMT-array gene expresion in PANC-1 cells after treatment with 9-cis-RA and TGFβ treatment; Table S7.Demographic details of the study participants; Figure S1. MRM MS analysis of acetyl CoA in human plasma. Panel (A). Representative chromatogram for acetyl CoA and the internal standard adenosine-2-D1. Panel (B). LC gradient used for acetyl CoA detection. Panels (C) and (D). A six point standard calibration curve for acetyl CoA; Figure S2. TGFβ treatment induced morphological changes in PANC-1 cells along with significant changes in the EMT markers (Panels A-D). Panel (A). Phase contrast images of PANC-1 cells treated with 10 ng/mL of TGFβ (right) and control cells (left). Panel (B). Bar graph showing significant down regulation in E- cadherin and concominant upregulation in N-cadherin. Panels (C) and D. Western blot analysis of altered levels of E-cadherin and N-cadherin in PANC-1 cells treated with 10 ng/mL of TGFβ (T) compared to control cells (C). TGFβ treatment did not induce EMT associated changes in Panc02.13 cells (Panels (E–H)). Panel €. Phase contrast images of Panc02.13 cells treated with 10ng/mL of TGFβ (right) and control cells (left). Panel (F). Bar graph showing statistically insignificant decrease in E-cadherin levels in Panc02.13 cells after TGFβ treatment. Panels (G, H). Western blot analysis of E-cadherin and N-cadherin in Panc02.13 cells treated with10 ng/mL of TGFβ (right) and control cells (left). Panel (I). Determination of endogenous levels of 9-cis-RA in PANC-1 and Panc02.13 by UPLC-MRM-MS. Panel (J). MS/MS validations of a subset of metabolites that were found to be significantly dysregulated in PANC-1 cells upon TGFβ treatment. Phase contrast (Panel (K)) and western blot (Panels (L, M) showing no change in morphology and EMT marker proteins upon (at) RA treatment; Figure S3. UPLC-MRM MS analysis of 9-cis retinoic acid in PANC-1 cells upon treatment with TGFβ. Panel (A). A representative chromatogram showing 9-cis RA peak. Panel (B,C). Description of LC-method used for resolution and detection of 9-cis RA. Panel (D). A six point standard calibration curve for 9-cis RA; Figure S4. Altered intracellular levels of collagen upon TGFβ and 9-cis RA treatment in PANC-1 cells. Western blot analysis of collagen protein levels in PANC-1 cells for the same set of treatments. Quantitative analysis was performed using BioRad Quantity One software. GAPDH was used for normalization. All *p* values were calculated using Student’s t test. *, *p* ≤ 0.05; Figure S5. In Vitro scratch assay showing the effect of TGFβ and 9-Cis RA treatment on PANC-1 and AsPC-1 cells. Cell migration images of scratch assay at 0, 24 and 48 h and bar graph presenting the wound area closure % based on scratch assay experiment for PANC-1 (Panel A) and AsPC-1 cells (Panel B); Figure S6. Correlation analysis suggesting moderate correlation between FN1 levels (as estimated by ELISA) and time of progression of PDAC. Patients with late progression tend to show lower plasma FN1 levels; Supplementary methods: RNA extraction and cDNA transcription; Real Time-Polymerase Chain Reaction (RT-PCR) on EMT related genes; Western blot analysis; 4 Scratch Assay; 9-cis RA MRM analysis in PANC-1 cells; MRM-MS analysis of amino acids; Validation of metabolites by targeted analysis in plasma samples; Figure S7. Primary Data for Supplementary Figure S2, panel C. References [21,60] are cited in the supplementary materials.
