# Supplementary material for: TGFβ Drives Metabolic Perturbations during Epithelial Mesenchymal Transition in Pancreatic Cancer: TGFβ Induced EMT in PDAC
Source: Cancers (Basel). 2021 Dec 9;13(24):6204. doi: 10.3390/cancers13246204 (PMC8699757; doi:10.3390/cancers13246204)
Supplement: Supplementary file 1 [file cancers-13-06204-s001.zip › cancers-1426308-supplementary.pdf]

Supplementary materials

TGFβ Drives Metabolic Perturbations during Epithelial Mesenchymal Transition in Pancreatic Cancer: TGFβ Induced EMT in PDAC

Meena U. Rajagopal, Shivani Bansal, Prabhjit Kaur, Shreyans K. Jain, Tatiana Altadil, Charles P. Hinzman, Yaoxiang Li, Joanna Moulton, Baldev Singh, Sunil Bansal, Siddheshwar Kisan Chauthe, Rajbir Singh, Partha P. Banerjee, Mark Mapstone, Massimo S. Fiandaca, Howard J. Federoff, Keith Unger, ill Smith and Amrita K. Cheema

Table S1. Statistical analysis of untargeted profiling data comparing PANC-1 cells treated with either TGFβ or DMSO. Up (green up arrow) or down (red down arrow), regulation of that particular metabolite or lipid.

m/z_rt	Mode	Fold Change (TGFβ/control)	log2(FC)	p.value	-log10(p)
296.0687_0.435	Pos	↓ 0.16607	-2.5902	8.84E-09	8.0538
258.1075_0.4803	Pos	↓ 0.22128	-2.176	3.69E-07	6.4331
156.0757_0.4544	Pos	↓ 0.24367	-2.037	7.87E-06	5.1043
249.0518_0.4514	Pos	↓ 0.29591	-1.7568	1.80E-05	4.7439
136.0751_0.527	Pos	↓ 0.23503	-2.0891	1.83E-05	4.7365
118.087_0.4839	Pos	↓ 0.35502	-1.494	3.27E-05	4.4849
148.0613_0.4811	Pos	↓ 0.35525	-1.4931	5.52E-05	4.2582
182.0803_0.5316	Pos	↓ 0.21392	-2.2249	7.50E-05	4.1247
533.4908_9.9742	Pos	↑ 2.3083	1.2068	0.00024436	3.612
146.0602_1.4642	Pos	↓ 0.15239	-2.7142	0.00031384	3.5033
132.1015_0.5914	Pos	↓ 0.18614	-2.4255	0.00031946	3.4956
357.2979_8.2775	Pos	↑ 4.4859	2.1654	0.00035108	3.4546
294.1458_0.6497	Pos	↓ 0.15862	-2.6564	0.00053074	3.2751
356.0734_3.2743	Pos	↓ 0.095916	-3.3821	0.00058299	3.2343
159.092_1.4567	Pos	↓ 0.23575	-2.0847	0.00078667	3.1042
245.1836_2.5461	Pos	↓ 0.10035	-3.3169	0.00083215	3.0798
166.0866_0.8059	Pos	↓ 0.18126	-2.4638	0.00088138	3.0548
238.0873_11.5319	Pos	↑ 4.295	2.1027	0.0010286	2.9877
340.0513_0.469	Pos	↓ 0.46859	-1.0936	0.0011681	2.9325
690.5397_9.8908	Pos	↑ 2.2551	1.1732	0.0013408	2.8726
308.093_0.5205	Pos	↓ 0.27296	-1.8733	0.0028454	2.5458
149.0588_0.7987	Pos	↓ 0.30979	-1.6906	0.0028635	2.5431
179.0723_0.4547	Pos	↓ 0.28754	-1.7982	0.0037635	2.4244
538.5215_9.9984	Pos	↑ 2.6798	1.4221	0.0039303	2.4056
489.3579_6.7811	Pos	↓ 0.41328	-1.2748	0.0046807	2.3297
229.1551_1.373	Pos	↓ 0.1647	-2.6021	0.0052486	2.28
121.0858_0.804	Pos	↓ 0.34553	-1.5331	0.0067573	2.1702
300.289_6.2419	Pos	↑ 2.32	1.2141	0.0070793	2.15
175.1188_0.4396	Pos	↓ 0.14333	-2.8026	0.0092466	2.034
494.3244_6.1714	Pos	↑ 3.9183	1.9702	0.010677	1.9715
828.6794_10.1245	Pos	↑ 2.2202	1.1507	0.010824	1.9656

283.272_6.2684	Pos	↑ 2.2178	1.1492	0.011167	1.9521
516.3052_6.1722	Pos	↑ 2.6164	1.3876	0.015384	1.8129
674.5184_9.1825	Pos	↑ 2.2545	1.1728	0.022835	1.6414
650.6414_10.1813	Pos	↑ 2.3569	1.2369	0.028374	1.5471
353.0485_3.2719	Neg	↓ 0.011457	-6.4477	1.70E-11	10.77
258.1451_1.0373	Neg	↓ 0.051637	-4.2755	8.56E-10	9.0674
244.1293_1.0471	Neg	↓ 0.062445	-4.0013	2.73E-09	8.5644
259.1315_1.1816	Neg	↓ 0.10376	-3.2686	1.21E-08	7.9173
221.0925_1.5383	Neg	↓ 0.083677	-3.579	3.64E-08	7.4386
306.0762_0.5185	Neg	↓ 0.35239	-1.5047	3.95E-08	7.4039
251.1022_1.3762	Neg	↓ 0.15587	-2.6816	3.18E-07	6.4977
187.1079_1.1188	Neg	↓ 0.14109	-2.8254	2.18E-06	5.6614
203.0817_1.4524	Neg	↓ 0.16919	-2.5633	2.90E-06	5.5383
277.1548_2.8806	Neg	↓ 0.1123	-3.1546	6.91E-06	5.1603
271.0691_1.4515	Neg	↓ 0.1959	-2.3518	3.24E-05	4.4893
540.0504_0.5142	Neg	↓ 0.48286	-1.0503	0.0002233	3.6511
164.0709_0.8043	Neg	↓ 0.27744	-1.8498	0.0002899	3.5378
333.0497_1.4478	Neg	↓ 0.3532	-1.5014	0.0013728	2.8624
154.0613_0.4364	Neg	↓ 0.23487	-2.0901	0.0015746	2.8028
188.0348_0.5824	Neg	↓ 0.38685	-1.3701	0.0016429	2.7844
299.2576_8.8985	Neg	↑ 27.541	4.7835	0.0025282	2.5972
130.0861_0.6025	Neg	↓ 0.26197	-1.9325	0.0037651	2.4242
360.1399_0.5988	Neg	↓ 0.34372	-1.5407	0.0069518	2.1579
180.0656_0.5512	Neg	↓ 0.30484	-1.7139	0.011851	1.9262
148.0426_0.7323	Neg	↓ 0.36314	-1.4614	0.017304	1.7619
593.2703_5.9892	Neg	↑ 3.3855	1.7594	0.022935	1.6395
354.1465_8.0716	Neg	↑ 6.4597	2.6915	0.03528	1.4525
253.2166_8.0857	Neg	↑ 7.7662	2.9572	0.042866	1.3679

Table S2. Full list of tandem MS validated metabolites with details

Metabolite	m/z	Mode	Formula	Metabolite ID	*p*-value	CID fragments
Hypoxanthine	137.0470	Positive	C5H4N4O	HMDB00157	0.000018300000	94.04, 110.03, 119.03, 120.02
D-Glutamic acid	148.0612	Positive	C5H9NO4	HMDB03339	0.000055200000	84.08, 130.08
L-Arginine	175.1190	Positive	C6H14N4O2	HMDB03416	0.009250000000	70.06, 116.06, 130.09, 158.08
D-Tyrosine	182.0800	Positive	C9H11NO3	C06420	0.000075000000	77.03, 95.04, 119.04, 136.07, 165.05
Leucyl-leucine	245.1844	Positive	C12H24N2O3	C11332	0.000832000000	86.09, 132.09
Glutathione	308.0930	Positive	C10H17N3O6S	HMDB00125	0.002850000000	76.02, 84.04, 116.01, 162.02, 291.06
Retinoic acid	301.2150	Positive	C20H30O2	HMDB01852	0.002530000000	201.2, 123.07
D-Aspartic acid	132.0303	Negative	C4H7NO4	HMDB06483	0.000000000017	88.04, 115.00
L-Phenylalanine	164.0709	Negative	C9H11NO2	HMDB00159	0.000289000000	103.05, 147.04
D-Tryptophan	203.0817	Negative	C11H12N2O2	HMDB13609	0.000002900000	74.02, 116.050, 142.06, 159.09, 186.05
Pseudouridine	243.0618	Negative	C9H12N2O6	HMDB00767	0.000000002730	110.02, 140.03, 153.02
Prostaglandin E2	333.2057	Negative	C20H30O4	HMDB02710	0.003530000000	113.09, 175.11, 235.13, 271.20, 315.19
Cyclic ADP-ribose	540.0504	Negative	C15H21N5O13P2	C13050	0.000223000000	134.04, 158.92, 272.95, 328.04
UDP-N-acetylglucosamine	606.0745	Negative	C17H27N3O17P2	HMDB00290	0.002290000000	158,92, 176.93, 282.03, 384.98, 402.99

Table S3. Statistical analysis results for the amino acid quantitation by targeted MRM-MS using UPLC-TQS comparing PANC-1 cells treated either with TGFβ or DMSO. Up (green up arrow) or down (red down arrow), regulation of that particular metabolite or lipid.

Metabolite	*p* value	FDR		Fold Change (TGFβ/control)	log2(FC)
Gln	4.72E-05	0.0014149	↑	2.9811	1.5759
Glu	8.03E-04	0.012044	↑	1.5389	0.62186
Val	0.0029031	0.021742	↑	1.5017	0.58658
Ile	0.0033284	0.021742	↑	1.5307	0.61419
Taurine	0.0036237	0.021742	↑	1.778	0.83025
Arg	0.0080191	0.031408	↑	1.2871	0.36413
Leu	0.0082324	0.031408	↑	1.4078	0.49346
His	0.0083755	0.031408	↑	1.5471	0.62959
Tyr	0.010348	0.034495	↑	1.4493	0.53536
Phe	0.015956	0.047319	↑	1.4367	0.52278
Gly	0.01735	0.047319	↑	1.5906	0.66955
Pro	0.021	0.05093	↑	1.4255	0.51146
Met	0.022069	0.05093	↑	1.355	0.43829
Trp	0.025753	0.055186	↑	1.3925	0.47771
ADMA	0.1077	0.2154	↑	1.3382	0.42025
Lys	0.12339	0.23136	↑	1.3643	0.44815
Thr	0.14745	0.26021	↑	1.1783	0.23665
Ala	0.23097	0.38494	↑	1.1905	0.25157
Dopamine	0.25913	0.40838	↓	0.8178	-0.29019
Serotonin	0.29821	0.40838	↑	1.0058	0.0083819
Asn	0.29823	0.40838	↑	1.1611	0.2155
Sarcosine	0.29948	0.40838	↑	1.1421	0.19166
Ser	0.37419	0.47369	↑	1.0433	0.061218
Putrescine	0.37895	0.47369	↓	0.97103	-0.042418
Carnosine	0.4922	0.55746	↓	0.84525	-0.24255
Spermidine	0.50088	0.55746	↓	0.95456	-0.067088
Creatinine	0.50171	0.55746	↑	1.1873	0.2477
Asp	0.88612	0.94223	↑	1.0068	0.0098242
c4-OH-Pro	0.91082	0.94223	↓	0.77529	-0.36719
Cit	0.98536	0.98536	↑	1.0037	0.0052629

Table S4. List of significantly dysregulated targeted MRM-MS data using QTRAP 7500 comparing PANC-1 cells treated either with TGFβ or DMSO. Up (green up arrow) or down (red down arrow), regulation of that particular metabolite or lipid.

Metabolite_mode	*p* value	FDR	Fold Change (TGFβ/control)	log2(FC)
Malate_Neg	1.05E-06	2.47E-04	↑ 1.933	0.95084
Uridine 5-Diphosphate_Neg	1.06E-06	2.47E-04	↑ 1.7387	0.79797
2-Hydroxyglutarate_Neg	1.70E-06	2.63E-04	↑ 2.4267	1.279
O-Acetylserine_Neg	4.99E-06	5.81E-04	↑ 2.3889	1.2564
IDP_Neg	8.82E-06	7.25E-04	↑ 1.7239	0.78566
Guanosine Diphosphate_pos	9.35E-06	7.25E-04	↑ 1.5676	0.64859
UDP-Glucose_Neg	1.20E-05	7.47E-04	↑ 1.574	0.65442
N-Acetylalanine_Neg	1.28E-05	7.47E-04	↑ 1.5636	0.64487
UDP-Glucuronate_Neg	1.76E-05	9.07E-04	↑ 1.8561	0.89224
Proline_pos	2.05E-05	9.53E-04	↑ 1.5291	0.61272
ADP_Neg	2.75E-05	0.0011055	↑ 1.7683	0.82233
UDP-N-Acetylamino Sugars_Neg	2.85E-05	0.0011055	↑ 1.4365	0.52256
L-2-Hydroxyglutaric Acid_Neg	3.73E-05	0.0013339	↑ 2.7022	1.4341
Glutamate_pos	7.36E-05	0.0024461	↑ 1.8675	0.90114
Phenylpropiolic Acid_Neg	8.56E-05	0.0025711	↑ 1.7006	0.76604
Threonic Acid_Neg	8.85E-05	0.0025711	↑ 1.5477	0.63015
2-Ketoisovalerate_Neg	9.57E-05	0.0026059	↑ 1.3226	0.40342
Guanidineacetate_Neg	1.10E-04	0.0026059	↑ 1.7013	0.76664
L-Glutamic Acid_pos	1.11E-04	0.0026059	↑ 1.6645	0.73511
Uridine Diphosphate Glucose_Neg	1.14E-04	0.0026059	↑ 1.5165	0.60075
Oxoglutarate_Neg	1.18E-04	0.0026059	↑ 2.1598	1.1109
Lactate_Neg	1.38E-04	0.0029164	↑ 1.5093	0.59386
Uridine 5-Diphosphate_pos	1.45E-04	0.0029296	↑ 1.5156	0.59993
Isocitrate_Neg	2.18E-04	0.0042168	↑ 2.0851	1.0601
Fumarate_Neg	2.66E-04	0.0049473	↑ 1.4206	0.50651
CDP_pos	2.82E-04	0.0050382	↑ 1.5132	0.59756
Betaine_pos_1	3.07E-04	0.0051699	↑ 1.3802	0.46484
ADP_pos	3.11E-04	0.0051699	↑ 1.436	0.52209
Norepinephrine_pos	3.47E-04	0.0055613	↑ 1.5814	0.66119
Alpha-Ketoisovaleric Acid_pos	4.18E-04	0.0064752	↑ 1.2975	0.37577
N-Acetylglycine_Neg	4.39E-04	0.00659	↑ 1.5721	0.65269
Glycerophosphocholine_pos	4.99E-04	0.0072486	↑ 1.4772	0.5629
L-Tryptophan_Neg	6.13E-04	0.0086227	↑ 1.3285	0.40983
Phenylacetylglycine_pos	6.30E-04	0.0086227	↑ 1.3589	0.44245

Homocysteic Acid_Neg	6.79E-04	0.0090156	↑	1.8682	0.90166
2-Phosphoglycerate_pos	7.00E-04	0.0090156	↓	0.68932	-0.53674
dGDP_Neg	7.17E-04	0.0090156	↑	1.5765	0.65671
Carbamoyl Phosphates_Neg	7.51E-04	0.0091839	↑	1.6285	0.70357
Gluconate_Neg	8.10E-04	0.009657	↑	2.2283	1.1559
3-Hydroxypropionic Acid_Neg	8.49E-04	0.0098683	↑	1.4833	0.56884
TAG506_FA204NH4	2.28E-05	0.0099907	↓	0.46719	-1.0979
TAG505_FA204NH4	3.22E-05	0.0099907	↓	0.54417	-0.87786
Pantothenic Acid_Neg	9.07E-04	0.01029	↑	1.4459	0.53193
Acetylserine_pos	9.49E-04	0.010511	↑	1.5453	0.62789
DAG161_183NH4	5.53E-05	0.011438	↓	0.69896	-0.51673
Ascorbate_Neg	0.001221	0.013204	↑	1.806	0.85278
Phosphoenolpyruvate_Neg	0.0014108	0.01491	↓	0.80335	-0.31589
Deoxyadenosine Triphosphate_pos	0.0015563	0.015753	↑	1.4485	0.5346
UDP_pos	0.0015584	0.015753	↑	1.5316	0.61501
Alpha-Tocopherol_Neg	0.0016679	0.016501	↑	1.4737	0.55944
Glyceraldehyde_Neg	0.0018865	0.018275	↑	1.4471	0.53315
Oxalate_Neg	0.0020284	0.019139	↑	1.5304	0.61394
NADP_Neg	0.0020611	0.019139	↑	2.3418	1.2276
3-Methylamino-L-Alanine_pos_1	0.0020991	0.019139	↑	1.3049	0.38399
Erythronic Acid_Neg	0.002488	0.022248	↑	1.4637	0.54963
cGMP_pos	0.00261	0.022899	↑	1.3621	0.44587
Betaine_pos_2	0.0027864	0.023994	↑	1.2857	0.3625
Ribose_Neg	0.0028802	0.024351	↑	2.5699	1.3617
Maleic Acid_Neg	0.0031468	0.025848	↑	1.2952	0.37315
ADP-Glucose_pos	0.0031685	0.025848	↑	1.3514	0.43447
DAG161_182NH4	1.75E-04	0.027095	↓	0.75793	-0.39987
D-Glucose_Neg	0.0035755	0.02825	↑	1.6759	0.74493
XMP	0.0035844	0.02825	↓	0.58653	-0.76973
Indole-3-Propionate_Neg	0.0037033	0.028701	↑	1.5607	0.64217
Cresol-Sulfate_Neg	0.0047303	0.036059	↑	1.4304	0.51647
GTP_Neg	0.0055858	0.041296	↑	1.8377	0.87792
N-Acetyl-Aspartate_Neg	0.0055949	0.041296	↑	1.6317	0.7064
Creatine_pos	0.0057613	0.04186	↑	1.3472	0.42997
2-Oxobutanoate_Neg	0.0064735	0.045974	↑	1.2441	0.31512
Fructose_Neg	0.0065254	0.045974	↑	1.5087	0.59327
L-Glutamine_pos	0.0067669	0.046964	↑	2.0261	1.0187

Butylcarnitine_pos	0.0068958	0.047155	1.3317	0.41332
Pyridoxamine_pos	0.0071769	0.048366	1.8287	0.87081
NADP_pos	0.0075473	0.049943	2.0128	1.0092
XMP_pos	0.0076258	0.049943	0.57985	-0.78624
NMN_Neg	0.0084741	0.054729	1.575	0.65534
N-Acetylmethionine_Neg	0.009507	0.060558	1.2906	0.36808
Deoxyadenosine Triphosphate_Neg	0.009775	0.061412	1.6509	0.72325
Dimethylarginine_pos	0.0099052	0.061412	1.4378	0.52388
L-Lysine_pos	0.010338	0.063255	1.8923	0.92017
DAG182_226NH4	5.28E-04	0.064131	0.60264	-0.73064
TAG505_FA183NH4	6.21E-04	0.064131	0.64897	-0.62378
DAG161_202NH4	7.58E-04	0.067108	0.69206	-0.53103
TAG548_FA183NH4	9.39E-04	0.072736	0.56073	-0.83461
Arabitol_Neg	0.012114	0.073159	1.5396	0.62258
DAG161_204NH4	0.0012098	0.075264	0.63413	-0.65715
DAG140_204NH4	0.0012139	0.075264	0.67566	-0.56563
Glycine_Neg	0.013862	0.082637	1.2549	0.32762
CDP-Choline_pos	0.014494	0.085316	1.5664	0.64749
DAG182_183NH4	0.0015392	0.086097	0.64833	-0.6252
TAG505_FA140NH4	0.0016664	0.086097	0.65342	-0.61391
NADP_pos.1	0.014991	0.087134	1.7648	0.81948
Alpha-D-Glucose_Neg	0.015245	0.087517	1.7778	0.83009
Spermidine_pos	0.016234	0.09206	1.2114	0.27662
Glutaric Acid_Neg	0.016463	0.092231	1.2948	0.37278
Fructose 1,6-Bisphosphate_Neg	0.017164	0.095017	1.2831	0.35963
Glyoxylate_Neg	0.018052	0.098755	1.2854	0.36227
Tryptophan_pos	0.018348	0.099205	1.1268	0.17228
FFA182	0.0022041	0.10074	0.68032	-0.55572
TAG485_FA183NH4	0.0022748	0.10074	0.61801	-0.69429
Indole-2-Carboxylic Acid_Neg	0.019661	0.10398	1.2332	0.3024
L-Tryptophan_pos	0.019679	0.10398	1.1481	0.1993
Threitol_Neg	0.021058	0.11002	1.308	0.38738
Ureidopropionic Acid_Neg	0.021891	0.1131	1.1962	0.25844
Citicoline_pos	0.022229	0.11359	1.3914	0.4765
TAG484_FA204NH4	0.0030596	0.12591	0.5911	-0.75852
DAG161_226NH4	0.0033304	0.12591	0.70781	-0.49856
TAG505_FA205NH4	0.0035726	0.12591	0.71509	-0.4838

TAG504_FA203NH4	0.0036555	0.12591	0.65093	-0.61943
Valine_pos	0.025542	0.1278	1.1708	0.22753
Cis-Aconitate_Neg	0.025908	0.1278	2.1012	1.0712
Itaconic Acid_Neg	0.026027	0.1278	1.2139	0.27966
Oxaloacetate_Neg	0.026215	0.1278	1.1784	0.23681
Ethylmalonic Acid_Neg	0.026386	0.1278	1.3061	0.38521
N-Acetylornithine_pos	0.027704	0.13281	0.70053	-0.51349
Guanosine Monophosphate_pos	0.029685	0.14085	0.73014	-0.45375
Asparagine_pos	0.030255	0.1417	1.3099	0.38941
2-Ketohexanoic Acid_Neg	0.030473	0.1417	1.2656	0.33987
TAG484_FA140NH4	0.0045011	0.14311	0.69383	-0.52734
LPC182AcO	0.0046165	0.14311	0.5543	-0.85126
2-Aminopimelic Acid_Neg	0.031954	0.14679	1.2974	0.37567
2,3-Diphospho-D-Glyceric Acid_Neg	0.0322	0.14679	2.1822	1.1258
Indole-3-Acetic Acid_Neg	0.032606	0.1472	1.3314	0.413
Alloisoleucine_pos	0.033331	0.14903	1.2242	0.29184
Glutathione Reduced_Neg	0.033984	0.1505	1.2412	0.31173
TAG569_FA204NH4	0.0051428	0.15183	0.47586	-1.0714
NAD_pos_1	0.035146	0.15409	1.2737	0.34903
Octanoylcarnitine_pos	0.035695	0.15409	2.3385	1.2256
N-Glycyl-L-Proline_pos	0.035822	0.15409	0.30832	-1.6975
Pantothenate_pos	0.036121	0.15409	1.2958	0.37385
2-Hydroxybutyrate_Neg	0.037571	0.15882	1.18	0.23873
Inositol_Neg	0.038009	0.15923	1.2835	0.36006
CE240H	0.0056943	0.16048	1.6878	0.75511
TAG484_FA182NH4	0.0062159	0.16316	0.66809	-0.58188
PS170_182-H	0.0065178	0.16316	1.5423	0.62508
TAG510_FA170NH4	0.0067264	0.16316	0.72595	-0.46205
PS201_202-H	0.0068424	0.16316	0.5859	-0.77126
Xanthine_Neg	0.039609	0.16445	1.1528	0.20509
Phosphoglycerates_Neg	0.041386	0.17031	0.80855	-0.30659
Xanthylic Acid_Neg	0.042485	0.17157	0.70348	-0.50743
O-Acetylserine_pos	0.042706	0.17157	1.2456	0.31689
Serine_pos	0.042801	0.17157	0.5452	-0.87514
ADP-Ribose_pos	0.043533	0.17301	0.53615	-0.89929
Indole_pos	0.044076	0.17306	1.1352	0.18293
Sorbitol_Neg	0.044289	0.17306	1.2617	0.33542

DAG140_182NH4	0.0078325	0.17973	0.78856	-0.34271
TAG524_FA224NH4	0.008117	0.17973	0.50315	-0.99094
Thiamine Pyrophosphate_Neg	0.048188	0.18552	1.2941	0.37191
dCTP_Neg	0.048638	0.18552	2.2545	1.1728
2-Deoxyguanosine 5-Monophosphate	0.048674	0.18552	1.1422	0.19186
TAG505_FA161NH4	0.0087257	0.18655	0.73525	-0.4437
3-Indolepropionate_pos	0.049887	0.18723	1.1657	0.22122
Xanthine/Oxypurinol_Neg	0.049927	0.18723	1.1698	0.22623
TAG485_FA182NH4	0.0091734	0.18911	0.61407	-0.70353
TAG503_FA140NH4	0.0094554	0.18911	0.7532	-0.4089
TAG548_FA182NH4	0.010025	0.19072	0.72307	-0.46779
TAG505_FA182NH4	0.010151	0.19072	0.65053	-0.62032
DAG182_224NH4	0.011277	0.20564	0.62274	-0.6833
TAG484_FA161NH4	0.01248	0.22108	0.79095	-0.33833
PS160_226-H	0.013603	0.23427	1.398	0.48339
TAG463_FA182NH4	0.015398	0.24508	0.70399	-0.50637
CL 721_281.2	0.015452	0.24508	1.6729	0.74238
TAG524_FA203NH4	0.015712	0.24508	0.65788	-0.60411
TAG504_FA140NH4	0.016288	0.24508	0.69479	-0.52534
DAG160_183NH4	0.016291	0.24508	0.84177	-0.24849
TAG463_FA183NH4	0.016776	0.24508	0.74014	-0.43413
TAG492_FA160NH4	0.016998	0.24508	1.2207	0.28775
TAG480_FA180NH4	0.018104	0.25293	0.78718	-0.34524
TAG504_FA204NH4	0.018358	0.25293	0.66371	-0.59138
TAG545_FA224NH4	0.0209	0.28123	0.50686	-0.98033
DAG182_203NH4	0.022505	0.28123	0.66078	-0.59776
TAG484_FA181NH4	0.022553	0.28123	0.79523	-0.33056
PS150_224-H	0.0229	0.28123	1.5529	0.63495
FFA181	0.023691	0.28123	0.77597	-0.36593
PS180_182-H	0.024091	0.28123	1.563	0.64429
PS161_224-H	0.02424	0.28123	0.73395	-0.44626
TAG525_FA203NH4	0.024314	0.28123	0.64841	-0.62503
TAG470_FA140NH4	0.024494	0.28123	0.78418	-0.35075
TAG502_FA202NH4	0.02674	0.29186	0.71189	-0.49028
TAG526_FA161NH4	0.027392	0.29186	0.68367	-0.54864
TAG510_FA180NH4	0.027746	0.29186	0.79067	-0.33885
TAG500_FA180NH4	0.027981	0.29186	0.73046	-0.45313

CL 726_283.2	0.028061	0.29186	0.2794	-1.8396
TAG523_FA140NH4	0.028245	0.29186	0.64366	-0.63564
TAG503_FA203NH4	0.029282	0.29762	0.68714	-0.54133
TAG524_FA202NH4	0.0306	0.3027	0.69629	-0.52224
TAG490_FA160NH4	0.030759	0.3027	0.83497	-0.2602
PS170_204-H	0.03195	0.30951	1.5079	0.59253
TAG461_FA180NH4	0.032819	0.31028	0.82886	-0.2708
TAG470_FA160NH4	0.03358	0.31028	0.81859	-0.28879
TAG504_FA161NH4	0.033748	0.31028	0.82024	-0.28588
TAG442_FA182NH4	0.034031	0.31028	0.67009	-0.57758
TAG525_FA204NH4	0.034566	0.31028	0.66501	-0.58856
TAG462_FA182NH4	0.035032	0.31028	0.7376	-0.43909
TAG440_FA160NH4	0.036312	0.31709	0.844	-0.24469
TAG461_FA161NH4	0.036844	0.31727	0.89001	-0.16811
TAG525_FA182NH4	0.037571	0.31909	0.69675	-0.52129
TAG567_FA224NH4	0.038286	0.32078	0.42821	-1.2236
CE224H	0.039696	0.32815	1.3706	0.45479
TAG505_FA160NH4	0.041545	0.33347	0.78422	-0.35066
TAG463_FA140NH4	0.041779	0.33347	0.70811	-0.49796
TAG460_FA180NH4	0.042309	0.33347	0.82208	-0.28266
PS161_180-H	0.042763	0.33347	2.2399	1.1635
TAG526_FA205NH4	0.043029	0.33347	0.81384	-0.29718
TAG526_FA204NH4	0.044527	0.34082	0.64304	-0.63701
TAG440_FA140NH4	0.046872	0.35065	0.83605	-0.25834
TAG504_FA182NH4	0.046942	0.35065	0.83262	-0.26427
TAG526_FA183NH4	0.047859	0.35324	0.69882	-0.517
TAG460_FA140NH4	0.049022	0.35732	0.82279	-0.28141

Table S5. Q1/Q3 transitions for metabolites quantified using multiple reaction monitoring mass spectrometry (MRM-MS).

Metabolites	Parent ion (m/z) Q1	Daughter ion (m/z) Q3	Cone voltage(V)	Collision energy (V)	Ionization mode
All trans RA-d5	306.2800	162.1654	28	22	positive
		205.0300	66	14	positive
		201.2012	60	16	positive
Retinoic acid	301.3685	123.0716	60	16	positive
Adenosine-2-d^{1}	268.8800	137.12	86	6	positive
Acetyl CoA	810.2853	91.1313	30	40	positive

Table S6. The CT values for EMT-array gene expresion in PANC-1 cells after treatment with 9-cis-RA and TGFβ treatment.

well#	Gene	control	control	TGFβ	TGFβ	9-cis RA	9-cis RA
A01	AHNAK	23.72213	24.001171	25.373894	25.212965	23.900797	22.97089
A02	AKT1	24.716043	24.865946	25.470478	25.137167	24.909576	24.970018
A03	BMP1	27.309622	27.448784	27.274576	27.26457	27.48462	27.683554
A04	BMP2	32.46994	32.79787	32.643734	33.095387	32.451546	32.064922
A05	BMP7	30.487295	30.804655	31.95954	32.190575	31.377134	30.240705
A06	CALD1	26.156288	26.004108	25.705177	25.459684	26.527279	26.89988
A07	CAMK2N1	29.695908	29.167866	28.991713	29.680927	28.75873	28.88902
A08	CAV2	24.475311	24.378048	24.718864	24.662163	23.849594	24.304272
A09	CDH1	28.18994	27.206379	28.398748	28.585682	27.06908	26.30639
A10	CDH2	26.399382	26.585398	25.660276	25.62273	26.421291	25.925327
A12	COL3A1	30.22915	29.984396	29.128742	28.264496	29.757551	29.290737
B01	COL5A2	23.94546	24.080275	22.817299	22.944618	23.303005	23.828856
B02	CTNNB1	27.11158	26.886679	27.330053	26.872774	27.53634	26.638065
B03	DSC2	27.969933	27.90065	28.123047	28.117176	28.109028	27.456753
B04	DSP	26.635202	26.311153	26.4048	26.362173	26.357178	25.576382
B05	EGFR	23.309435	23.432787	23.955069	23.88893	23.176468	22.775122
B06	ERBB3	29.75765	30.072342	29.4883	29.32204	30.223972	29.83775
B07	ESR1	35.38256	35.266315	35.977093	36.593674	36.44503	35.39192
B08	F11R	25.162985	25.2715	25.454098	25.271702	25.363554	24.66876
B10	FN1	27.802143	27.636286	26.521307	26.250874	26.791447	26.098782
B11	FOXC2	27.471842	27.410688	27.45844	27.386799	27.61804	27.878096
B12	FZD7	28.010073	28.157347	28.229391	28.315681	27.858418	27.62633
C01	GNG11	30.144403	29.551682	30.435877	30.468008	29.399435	29.51467
C02	GSC	32.784836	32.825596	33.229156	32.82066	33.369858	32.293137
C03	GSK3B	26.468384	26.38953	26.322752	26.669819	26.124872	26.069475
C04	IGFBP4	26.428608	26.182045	26.723322	26.765253	26.571337	26.404688
C06	ILK	23.178232	23.165524	23.590351	23.301498	23.210276	23.3959
C07	ITGA5	24.90651	24.896955	24.507765	24.448751	24.163416	24.544832
C08	ITGAV	22.57352	22.566622	22.41121	21.903906	23.335123	22.81176
C09	ITGB1	22.089487	21.71333	21.848166	21.554403	21.333174	21.17167
C10	JAG1	24.949516	24.824884	23.216496	23.239279	24.621735	24.64482
C11	KRT14	32.846924	33.331963	38.011307	35.799213	30.87995	34.20887
C12	KRT19	23.91453	24.384045	26.00303	25.622774	21.863655	23.096504
D01	KRT7	32.959957	32.179035	32.598465	31.805792	31.840647	34.838634
D02	MAP1B	24.308073	24.283142	23.487259	23.467625	24.149122	23.396605
D03	MMP2	23.722553	23.669231	22.631474	22.716732	23.386957	23.008833
D05	MMP9	30.321085	30.172823	30.60738	30.709908	30.326618	30.858786
D06	MSN	23.823055	23.717724	23.746714	23.696815	24.27202	23.654661
D07	MST1R	25.66755	25.797096	27.308674	26.754816	26.135452	26.570486
D08	NODAL	30.184517	29.925932	30.650589	30.94477	30.743912	30.16004
D09	NOTCH1	29.133568	29.251791	28.579674	28.988064	29.39544	28.812618
D10	NUDT13	25.754204	25.52295	25.743847	25.671064	25.474466	26.212975
D11	OCLN	25.10093	24.91363	24.976164	25.293768	24.442993	24.282402
D12	PDGFRB	33.789474	33.715214	30.956247	30.67355	34.192135	34.86569
E01	PLEK2	29.314657	29.123709	27.348488	27.547367	28.716623	29.763626
E02	DESI1	25.322342	25.49086	25.41833	25.280693	24.865448	25.15678
E03	PTK2	24.552269	24.305845	24.347153	24.549002	23.99211	24.598883
E04	PTP4A1	24.971855	24.707754	24.316957	24.328968	24.836185	24.757366
E05	RAC1	22.771023	22.571993	22.91725	22.822977	22.48092	22.745274
E06	RGS2	28.59829	28.598232	28.889122	29.477032	27.578999	29.208578
E07	SERPINE1	21.273634	21.221365	18.940994	19.192759	20.664064	20.552711
E08	GEMIN2	27.330616	26.857687	26.417065	26.556849	26.587574	27.403286
E09	SMAD2	24.984016	24.861345	25.254725	25.188396	24.939096	24.681183
E10	SNAI1	31.754438	31.54407	29.888979	29.862576	32.309776	32.317303

E12	SNAI3	27.613657	27.798435	28.586746	28.146723	27.995163	27.757162
F02	SPARC	26.958439	27.009361	27.700678	27.5204	25.232622	24.786722
F04	STAT3	22.963017	23.128721	23.868423	23.802593	22.776419	22.542494
F05	STEAP1	26.230211	26.29684	26.421646	26.039219	25.36733	26.79008
F06	TCF3	25.463226	25.471003	25.473618	25.62543	25.030828	25.859816
F07	TCF4	28.578587	28.450489	27.946072	28.774704	27.939846	28.130228
F08	TFPI2	30.927755	30.99183	31.330347	28.504354	29.178204	29.134922
F10	TGFB2	24.315224	24.106289	24.314777	24.126863	23.533129	23.864262
F11	TGFB3	27.665234	27.822187	28.630426	28.210752	27.76456	27.667475
F12	TIMP1	25.940151	27.77124	27.915367	27.92035	27.651844	28.528038
G02	TMEM132A	24.903408	24.888134	25.667484	24.674028	24.576675	24.406626
G03	TSPAN13	24.099522	24.378971	25.367882	25.408718	24.450918	24.786058
G05	VCAN	29.660183	30.876116	29.277136	28.870779	30.641521	30.269152
G06	VIM	19.961771	19.880146	19.437693	19.340944	19.701712	19.684406
G07	VPS13A	24.111542	23.843824	24.310503	24.322866	23.547522	24.287523
G08	WNT11	26.171305	31.721075	31.853115	29.451378	33.64326	32.003315
G09	WNT5A	27.981655	30.150057	29.009012	29.492409	29.698029	30.832056
G10	WNT5B	26.28062	26.589397	26.613243	26.726038	26.43868	26.52162
G11	ZEB1	26.96873	26.792606	26.948341	26.692385	26.871046	26.971975
H01	ACTB	20.027222	20.180428	19.69842	20.032513	19.837532	19.780613
H02	B2M	22.753914	22.714182	22.981619	23.010551	21.68105	22.600576
H03	GAPDH	22.21239	22.141045	22.474829	22.35109	21.570358	21.267801
H04	HPRT1	26.512363	26.49413	26.182943	26.419352	26.358446	26.475296
H05	RPLP0	20.239859	20.205614	20.27759	20.128023	19.63809	19.450907
H07	RTC	22.093557	21.981878	21.500277	22.48454	21.739742	22.264055
H08	RTC	21.776161	21.874844	21.597733	22.374355	21.659988	22.069073
H09	RTC	21.97254	21.913893	21.440775	22.838408	21.829437	22.302492
H10	PPC	19.202522	20.501808	20.339687	20.055498	19.883312	20.304695

Table S7. Demographic details of the study participants.

	**Normal (n= 9)**	**Pancreatitis (n=10)**	**IPMN (n=9)**	**PDAC (n=11)**
Median age (years)	80.4	57.2	53.7	60.9
		Ethnicity		
Caucasian	9	6	7	9
African American	0	2	1	2
Asian	0	1	0	0
Hispanic	0	0	1	0
Other	0	1	0	0
		Gender		
Male	6	7	1	6
Female	3	3	8	5
Type II Diabetes	2	3	2	1
Mean BMI	26.6	25.6	30.2	29.1
Alcohol (YES)	9	3	4	5
Smoking (YES)	0	4	6	4
Jaundice (YES)	0	0	3	4

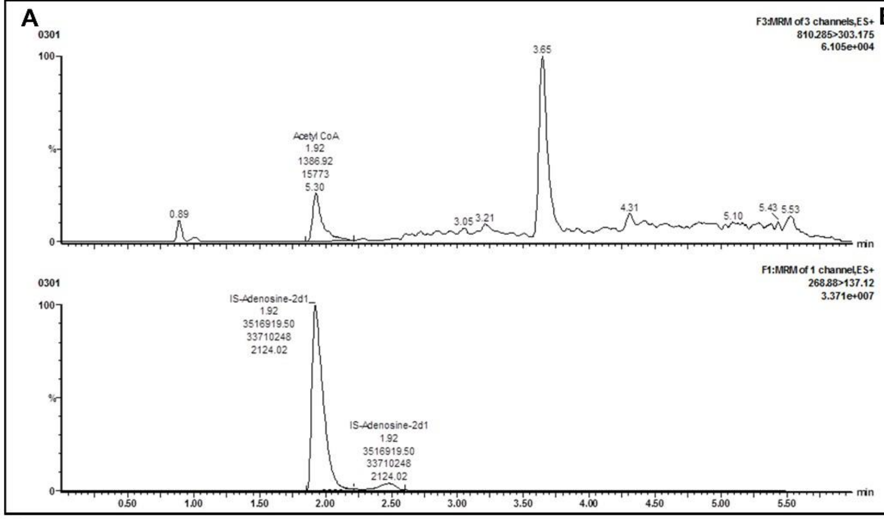

B **LC method:**

Mobile Phase

Solvent A: ammonium acetate (10mM) + ammonium carbonate (5mM)

Solvent B: ACN + 0.2% formic acid

Column: ACQUITY UPLC BEH C18 1.7µm X 50mm

Column Temp: 40 °C.

Time (min)	Flow rate(mL/min)	% A	% B
0	0.25	100	0
3	0.25	20	80
3.5	0.25	0	0
4	0.25	0	0
5	0.25	100	0
5.5	0.25	100	0

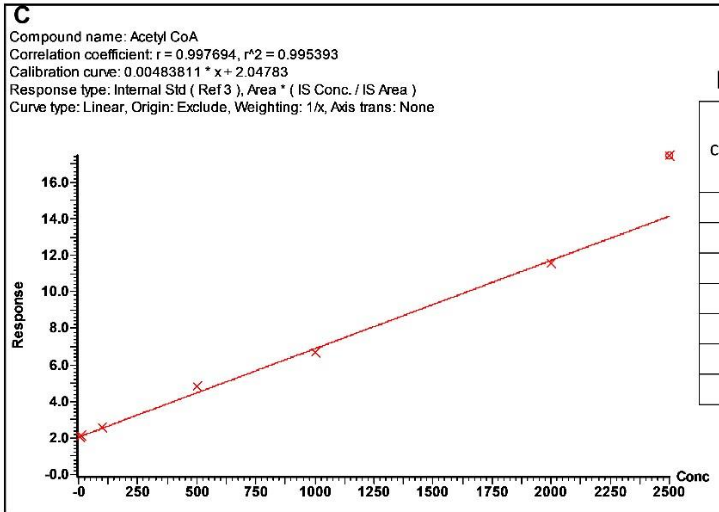

D

Standard concentration (ng/mL)	RT (min)	Area	IS Area	Response	Concentration (ng/mL)	% Dev
5	1.85	908278	439051.4	2.069	4.3	-13.6
10	1.85	950488.9	453824.7	2.094	9.6	-3.8
100	1.85	1142828	444379.9	2.572	108.3	8.3
500	1.86	2305917	475633.6	4.848	578.8	15.8
1000	1.86	3461103	519942.2	6.657	952.6	-4.7
2000	1.85	6199525	537355.1	11.537	1961.4	-1.9
2500	1.87	12520494	716621.8	17.472	3188	27.5

Figure S1. MRM MS analysis of acetyl CoA in human plasma. Panel(A). Representative chromatogram for acetyl CoA and the internal standard adenosine-2- D1. Panel (B). LC gradient used for acetyl CoA detection. Panels (C, D). A six point standard calibration curve for acetyl CoA

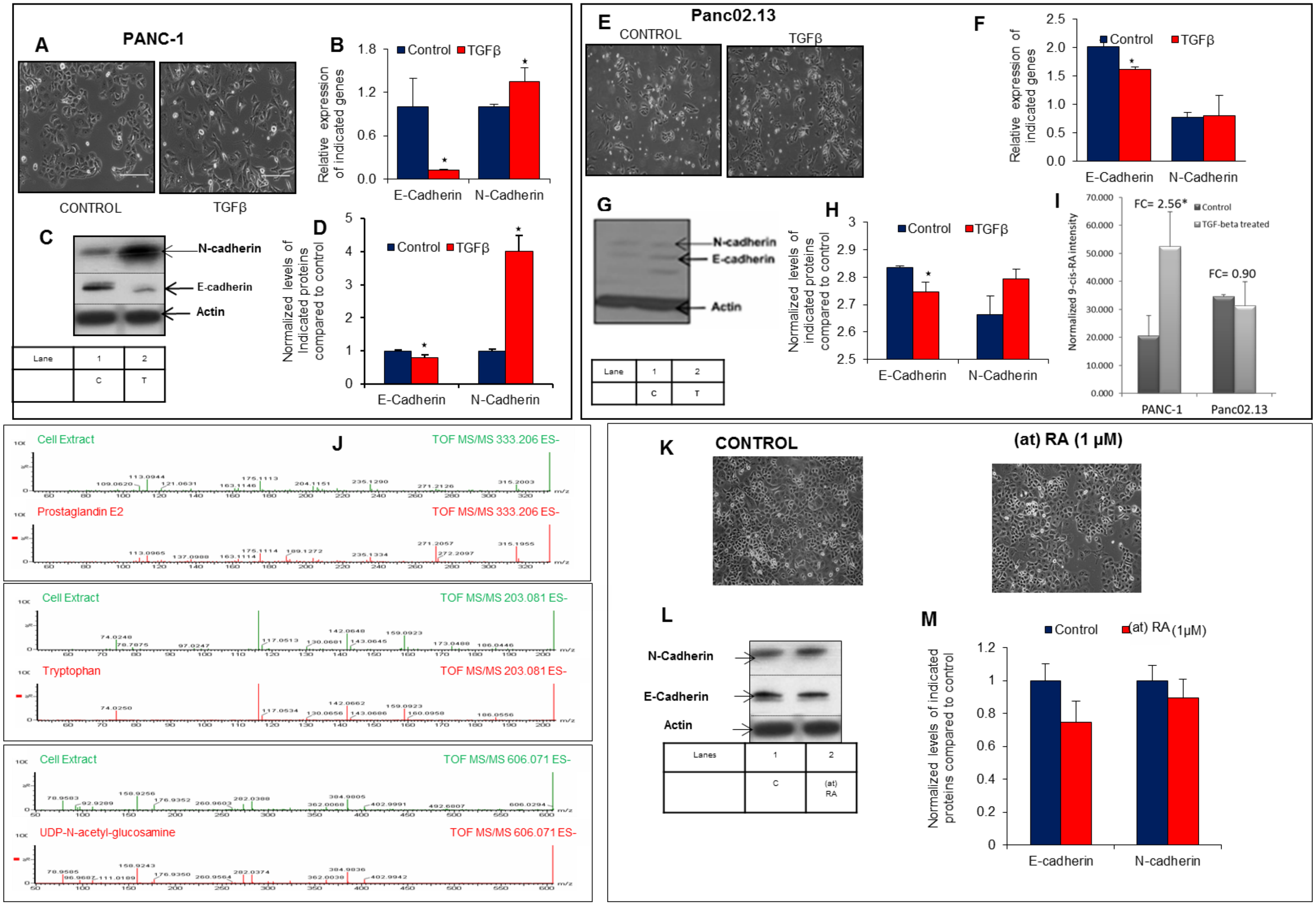

Figure S2. TGFβ treatment induced morphological changes in PANC-1 cells along with significant changes in the EMT markers (Panels A-D). Panel (A). Phase contrast images of PANC-1 cells treated with 10 ng/mL of TGFβ (right) and control cells (left). Panel (B). Bar graph showing significant down regulation in E- cadherin and concomitant upregulation in N-cadherin. Panels (C, D). Western blot analysis of altered levels of E-cadherin and N-cadherin in PANC-1 cells treated with 10 ng/mL of TGFβ (T) compared to control cells (C). TGFβ treatment did not induce EMT associated changes in Panc02.13 cells (Panels E-H). Panel E. Phase contrast images of Panc02.13 cells treated with 10ng/mL of TGFβ (right) and control cells (left). Panel F. Bar graph showing statistically insignificant decrease in E-cadherin levels in Panc02.13 cells after TGFβ treatment. Panels G and H. Western blot analysis of E-cadherin and N-

cadherin in Panc02.13 cells treated with10 ng/mL of TGFβ (right) and control cells (left). Panel I. Determination of endogenous levels of 9-cis-RA in PANC-1 and Panc02.13 by UPLC-MRM-MS. Panel J. MS/MS validations of a subset of metabolites that were found to be significantly dysregulated in PANC-1 cells upon TGFβ treatment. Phase contrast (Panel K) and western blot (Panels L and.M) showing no change in morphology and EMT marker proteins upon (at) RA treatment.

A

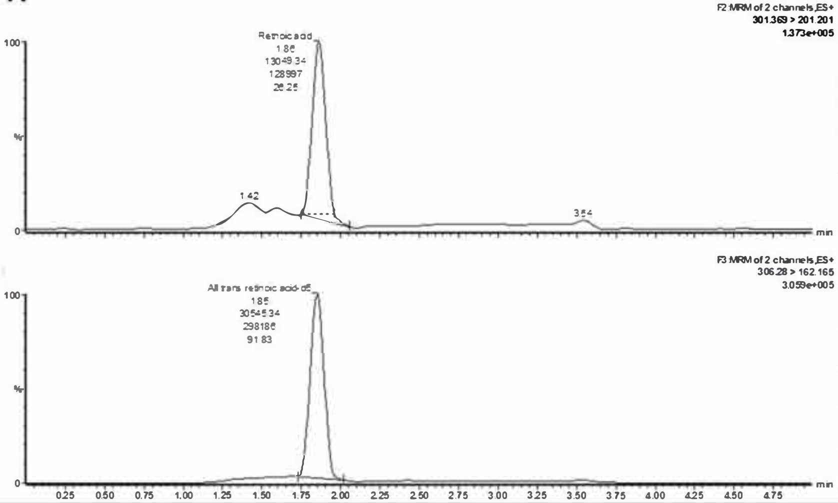

B

Mobile Phase

Solvent A: Water + 0.2% formic acid

Solvent B: MeOH + 0.1% formic acid

Column: ACQUITY UPLC BEH C18.1.7μm, 2.1mm X 100mm

Column Temp: 40 °C.

**Time (min)**	**Flow rate(mL/min)**	**% A**	**% B**
Initial	0.400	95	5
0.5	0.400	95	5
1.0	0.400	1	99
3.0	0.400	1	99
3.25	0.400	95	5
5.0	0.400	95	5

C

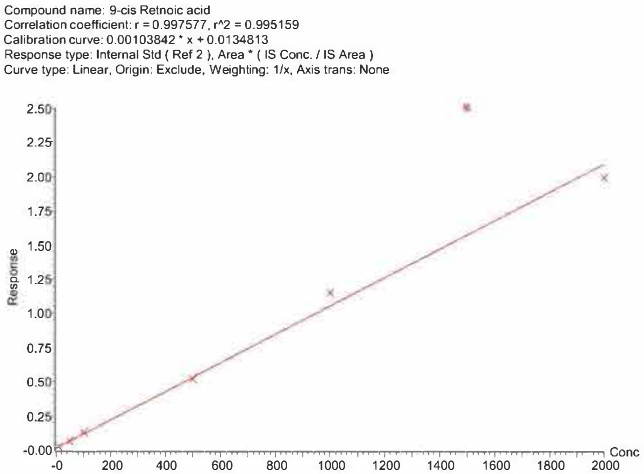

D

Standard Concentration (ng/mL)	RT (min)	Area	IS Area	Response	Concentration (ng/mL)	%Dev
10	2.25	1165.275	53468.293	0.022	8	-20
50	2.25	4296.439	64145	0.067	51.5	3
100	2.25	4706.847	35604.598	0.132	114.3	14.3
500	2.25	27577.623	52761.523	0.523	490.4	-1.9
1000	2.25	53890.547	46858.906	1.15	1094.5	9.5
2000	2.25	125557.117	63164.113	1.988	1901.3	-4.9

Figure S3. UPLC-MRM MS analysis of 9-cis retinoic acid in PANC-1 cells upon treatment with TGFβ. Panel A. A representative chromatogram showing 9-cis RA peak. Panel B-C. Description of LC-method used for resolution and detection of 9-cis RA. Panel D. A six point standard calibration curve for 9-cis RA.

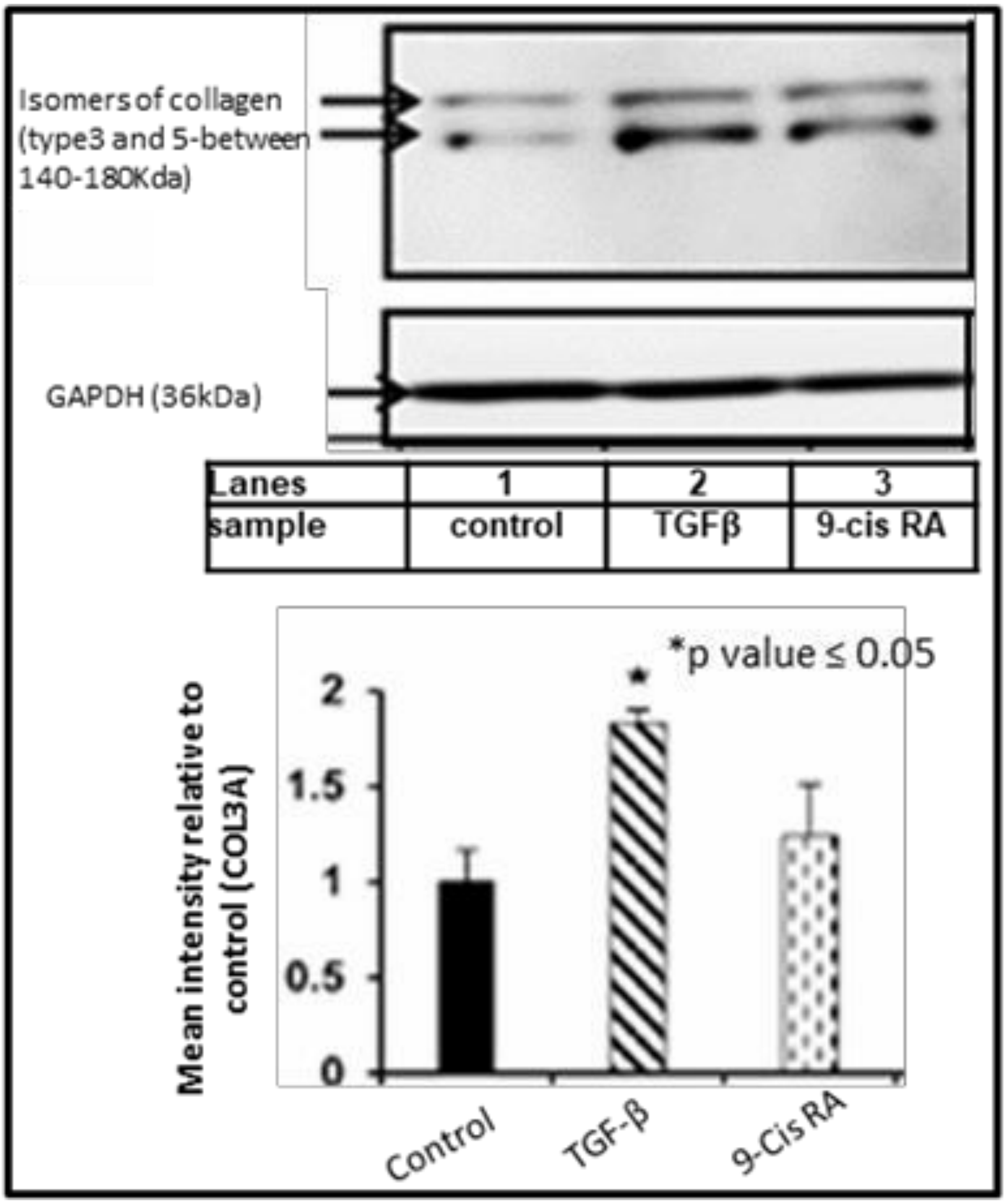

Figure S4. Altered intracellular levels of collagen upon TGFβ and 9- Cis RA treatment in PANC-1 cells. Western blot analysis of collagen protein levels in PANC-1 cells for the same set of treatments. Quantitative analysis was performed using BioRad Quantity One software. GAPDH was used for normalization. All p values were calculated using Student's t test. *, $p \leq 0.05$.

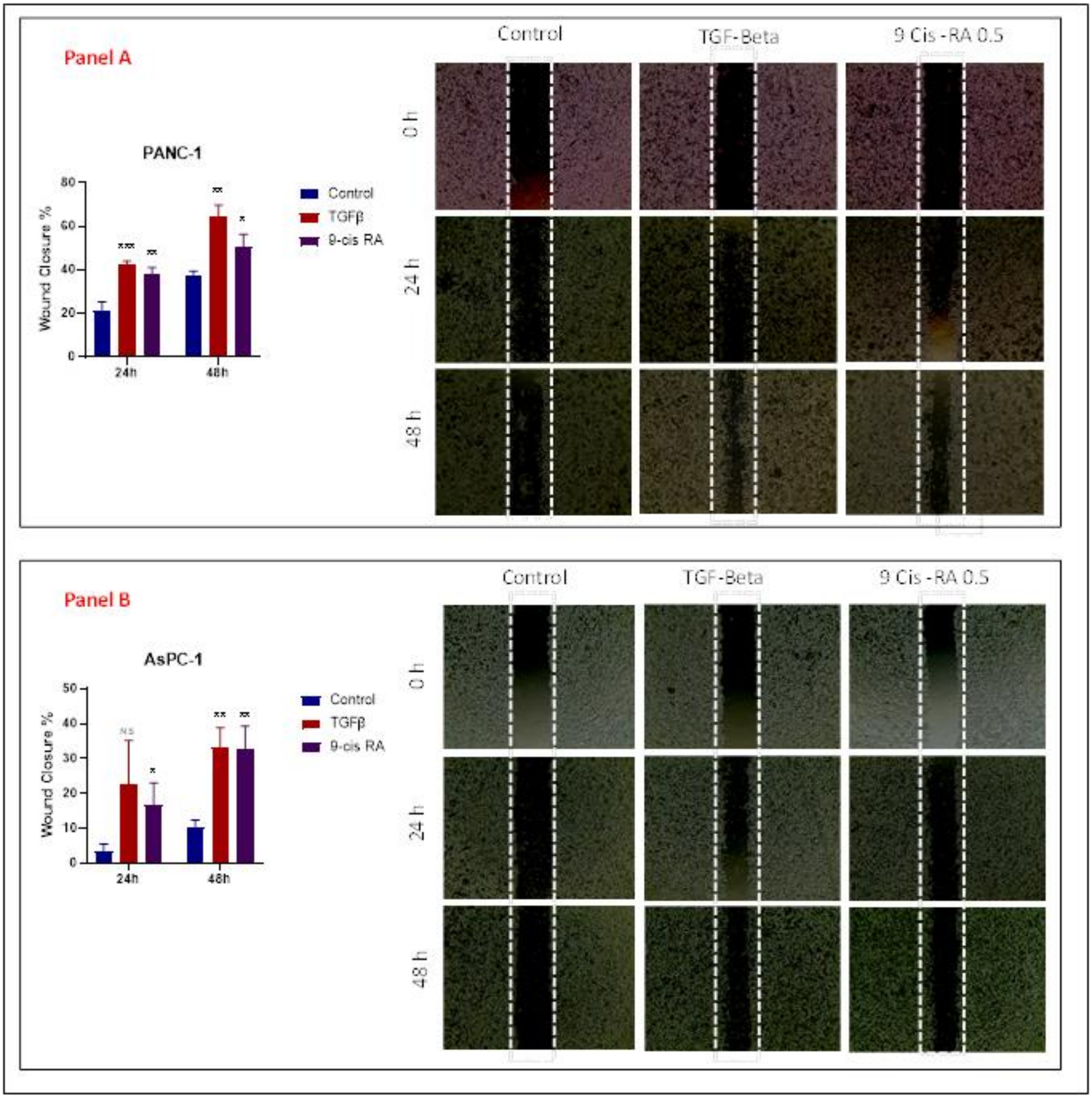

Figure S5. In Vitro scratch assay showing the effect of TGFβ and 9-Cis RA treatment on PANC-1 and AsPC-1 cells. Cell migration images of scratch assay at 0, 24 and 48 h and bar graph presenting the wound area closure % based on scratch assay experiment for PANC-1 (Panel A) and AsPC-1 cells (Panel B).

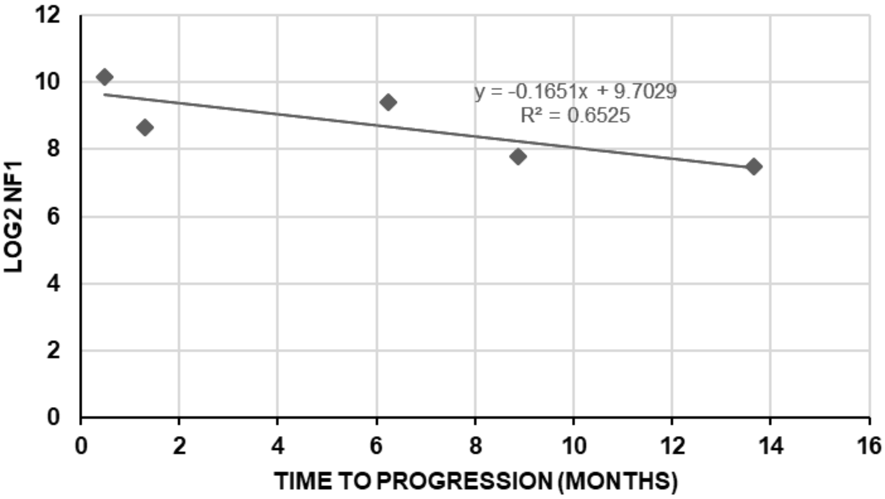

Figure S6. Correlation analysis suggesting moderate correlation between FN1 levels (as estimated by ELISA) and time of progression of PDAC. Patients with late progression tend to show lower plasma FN1 levels.

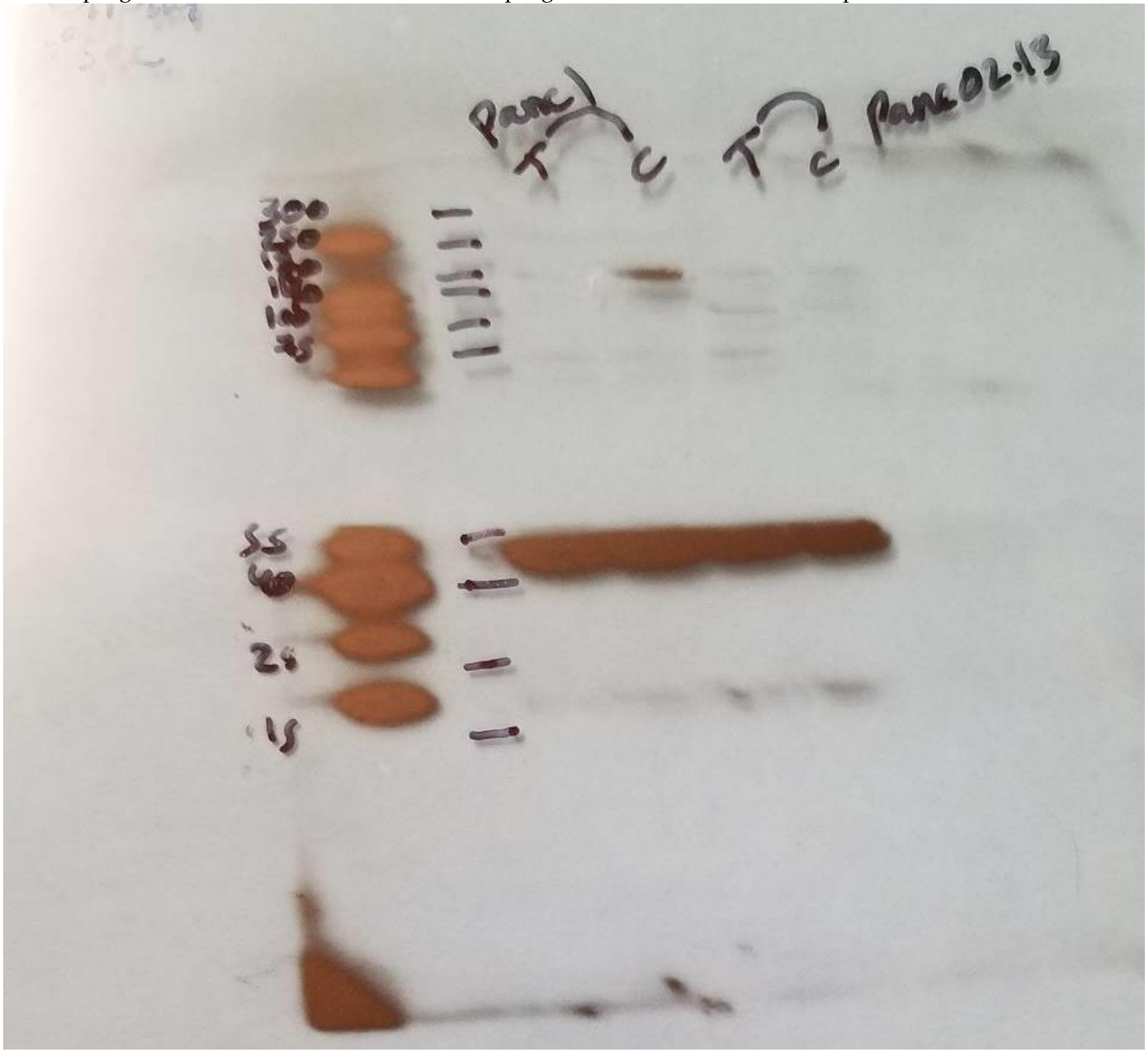

Figure S7. Primary Data for Supplementary Figure S2, panel C

Supplementary Methods

RNA extraction and cDNA transcription

Total RNA was isolated from PANC-1 cells using RNeasy Mini kit (Qiagen, Hilden, Germany) as per standard protocol provided by the manufacturer, with on-column DNA digestion. RNA integrity and concentration was analyzed using nanodrop ND-1000 v 3.8.1 spectrophotometer. OD 260/280 values were between 2.1 and 2.4. Total RNA (100 ng) was used to obtain cDNA. Reverse-Transcription Reaction was performed following the standard protocol of RT^2 First Strand Kit (Qiagen, CA, USA). The cDNA obtained was analyzed by real time PCR using ABI 7900HT.

Real Time-Polymerase Chain Reaction (RT-PCR) on EMT related genes

To examine the effect of TGFβ and 9-cis RA on the expression of genes related to EMT, we used an RT^2 Profiler PCR Array for human EMT (Qiagen) consisting of quantitative RT-PCR of 84 EMT-related genes. The primers for FN1 (PPH001438-200), Col3A (PPH00439F-200), OGT (PPH01166A-200) and GAPDH (PPH00150F-200) were procured from Qiagen. Real-time PCR experiments were conducted using the SYBR Green PCR system (Applied Biosystems, Foster City, CA), with 40 cycles per sample. Cycling temperatures were as follows: 95°C for 10 min, 40 cycles at 95°C for 15 secs and then 60°C for 1 min. Data were analyzed using $2^{-\Delta\Delta Ct}$ method.

Western blot analysis

Cells were lysed in RIPA buffer (Alfa Aesar) and total protein was quantified using detergent compatible Bradford assay reagent (Pierce). 50 µg of total protein from control (no treatments) and treated samples were loaded on to Novex Wedge well 4-12% Tris glycine polyacrylamide gel (Invitrogen) and electrophoresis was carried out at constant 100V until the blue dye reached the bottom of the gel. Proteins were transferred onto PVDF membrane at constant 15V overnight in the cold room. Membranes were blocked with 5% non-fat milk (Lab Scientific Inc.) in PBS for 1 hour. All primary antibodies (Abcam- rabbit polyclonal human E-cadherin (1:1000), rabbit polyclonal human N-cadherin (1:1500), rabbit polyclonal human fibronectin was used at 1:1250 dilution while rabbit polyclonal human collagen III was used at 1:1000 dilution) were prepared in blocking solution and incubated overnight at 4 ºC. The membranes were washed and incubated with horseradish peroxidase (HRP) - conjugated secondary antibodies (goat anti rabbit IgG-HRP conjugate, Invitrogen) at 1:10,000 dilution in TTBS for 2 hours at room temperature. GAPDH was used as a loading control (rabbit polyclonal human GAPDH from Abcam) at 1:2500 dilution in TTBS solution. Finally, the membranes were washed and developed with enhanced chemiluminescence reagents (Amersham Corporation) and visualized using GE Amersham Imager 600. Densitometry was performed using Quantity One (BioRad) by obtaining the optical density of each band.

Scratch Assay

The cells were seeded in 12 well cell culture treated plates and allowed to grow until become 80-90% confluent followed by starvation for 24 h in FBS depleted medium. Following starvation, the scratch was made using 200 µl pipette tip, cells were washed with PBS and TGF and 9-cis RA treatments were given fresh FBS depleted medium. The images were captured at 0, 24 and 48 h after treatments. The wound area was calculated using ImageJ software (https://imagej.nih.gov/ij/) and wound closure% was calculated using the following mentioned formula [1].

$$Wound\ closure\% = \left(\frac{(Area\ at\ 0h - Area\ at\ (24\ or\ 48\ h) after\ treatment)}{Area\ at\ 0h}\right) \times 100\%$$

9-cis RA MRM analysis in PANC-1 cells

Metabolite extraction:

Metabolite extraction was performed as per the protocol explained by Kane et al [2]. The cell pellets were sonicated in 25 µL of water. Deuterium labelled all trans retinoic acid (Santa Cruz Biotechnology Inc, TX, USA) was used at 300 ng/mL as internal standard. 1 mL of 0.025 M KOH in ethanol was added to the homogenate and vortex for 10 sec. ACN (1mL) was added and vortex for at least 10 sec. Addition of 10 mL of hexane was followed by the addition of 60 µL of 4 M HCl and the mixture was vortex well. The mixture was centrifuged for 3 min at 1000 X g to facilitate phase separation. Top organic layer containing 9-cis RA was separated and evaporated under a gentle

stream of nitrogen and re-suspended in 200 μL of 50:50 methanol:water.

Standards preparation and quantitation:

A stock solution of D5-(at) RA (internal standard) and 9-cis retinoic acid were prepared in 90:10 of methanol:water at a concentration of 1 μg/mL. Calibration curve solutions with concentrations from 10 ng/mL to 2000 ng/mL of were prepared from the stock solutions.

LC-MRM MS conditions:

The extracted metabolites were resolved on a BEH C18 column using the gradient method shown in Supplementary Figure S3, panel B. A binary solvent comprising of water (with 0.2% formic acid) and methanol (with 0.2% formic acid) was used. MRM transitions that were used for quantitation are detailed in Supplementary Table S5. The data were normalized to total protein content and processed using Target Lynx 4.1 available within the Mass Lynx software suite (Waters Corporation).

MRM-MS analysis of amino acids

As indicated in the main text, intra cellular amino acids were extracted from PANC-1 cell pellets after various treatments and subjected to derivatization using phenylisothiocyanate (PITC) using commercially available protocol (Biocrates Absolute IDQ p180 kit). The Kit contains 7 calibrants, 3 QCs and an Internal standard, as solid lyophilized powders. The solutions were made as per the instructions of Biocrates module. 100 μL of HPLC grade water was added to each of the seven calibrants (KIT2 cal1-cal7) and 3 QCs. Added 1200 μL of HPLC grade water to lyophilized Internal standard. Shaken all the vials for 15 minutes at 1200 rpm and vortexed several times. Took 10 μL from stock solution of respective vial (cell pellet as well) and added 10 μl of internal standard stock solution. Dried under nitrogen evaporator. To this added, 50 μL of 5% v/v PITC solution. Vortexed and sonicated for 5 min and kept standing for 20 min. Dried the solution using nitrogen evaporator. Added 300 μL of extraction buffer to every vial. Shaken it well for 20 min. Taken 150 μL of this solution and diluted it with 150 μL of water solution. Transferred to the mass spec vial and run the sample.

Validation of metabolites by targeted analysis in plasma samples

Plasma samples were prepared as discussed under methods section in the main manuscript. Standards (acetyl CoA) were prepared at 1 μg/mL concentration. Stock solutions of internal standards (adenosine-2-d2) were also prepared at 1 μg/mL concentration. The LC gradient method along with the standard calibration curve was generated with at least 6 different concentrations of the standard as shown in Supplementary Figure S1. MRM transitions are detailed in Supplementary Table S5.
